# Transcriptome Response to Cadmium Exposure in Barley (*Hordeum vulgare* L.)

**DOI:** 10.3389/fpls.2021.629089

**Published:** 2021-07-15

**Authors:** Martina Kintlová, Jan Vrána, Roman Hobza, Nicolas Blavet, Vojtěch Hudzieczek

**Affiliations:** ^1^Czech Academy of Sciences, Centre of the Region Haná for Biotechnological and Agricultural Research, Institute of Experimental Botany, Olomouc, Czechia; ^2^Czech Academy of Sciences, Institute of Biophysics, Brno, Czechia

**Keywords:** barley, cadmium, transcriptomic analysis, *HvPCR2*, gene duplication

## Abstract

Cadmium is an environmental pollutant with high toxicity that negatively affects plant growth and development. To understand the molecular mechanisms of plant response to cadmium stress, we have performed a genome-wide transcriptome analysis on barley plants treated with an increased concentration of cadmium. Differential gene expression analysis revealed 10,282 deregulated transcripts present in the roots and 7,104 in the shoots. Among them, we identified genes related to reactive oxygen species metabolism, cell wall formation and maintenance, ion membrane transport and stress response. One of the most upregulated genes was *PLANT CADMIUM RESISTACE 2 (HvPCR2)* known to be responsible for heavy metal detoxification in plants. Surprisingly, in the transcriptomic data we identified four different copies of the *HvPCR2* gene with a specific pattern of upregulation in individual tissues. Heterologous expression of all five barley copies in a Cd-sensitive yeast mutant restored cadmium resistance. In addition, four *HvPCR2* were located in tandem arrangement in a single genomic region of the barley 5H chromosome. To our knowledge, this is the first example showing multiplication of the *PCR2* gene in plants.

## Introduction

Cadmium (Cd) is a particularly dangerous environmental pollutant with a high toxicity to plants and other living organisms, including humans. Its concentration increases rapidly in the environment as an effect of anthropogenic factors such as mining, metal-work industries, urban traffic or the application of phosphate fertilizers (Nriagu and Pacyna, 1988). Cadmium contamination of soils can cause losses in agricultural yield and present a potential health risk for people from Cd transfer through the food chain.

Cadmium has no known function as a nutrient and its uptake by plants is dependent on soil concentration, bioavailability, redox potential, temperature, quantity of organic matter, and concentrations of other elements. Cd accumulation in plants affects various processes such as water and mineral uptake, photosynthesis and respiration, resulting in inhibition of growth and even death (Sanità Di Toppi and Gabbrielli, 1999). Cd ions compete with other nutrients for plant uptake and enter the plant via Fe^2+^/Fe^3+^, Zn^2+^, and Mn^2+^ transporters because of their limited specificity (Pedas et al., 2008; Sasaki et al., 2012). Thus, the plant damage caused by cadmium and other heavy metals comes from competition with essential mineral nutrients leading to deficient nutrition (Clarkson and Lüttge, 1989). Once Cd enters the root, it can be sequestrated in root vacuoles or translocated in the xylem through the apoplastic and/or symplastic pathway and transported into the aboveground parts of the plant (Salt and Rauser, 1995; Yang et al., 1998).

In addition, heavy metals may accumulate in cell compartments that impede the general metabolism of the plant (Thurman and Collins, 1983). As a response to biotic or abiotic stress, plants produce common stress-related transcripts like transcriptional factors, transporter proteins and other genes involved in signal transduction and secondary metabolite pathways (Atkinson and Urwin, 2012). Plants have developed a complex network of mechanisms to minimize the toxic effect of cadmium import. These active and passive strategies based on exclusion of the Cd^2+^ from the cellular environment are applied at several levels. As a first response, malate or citrate is secreted and binds to metal ions thereby restricting Cd root absorption (Delhaize and Ryan, 1995). If Cd is absorbed, it can be immobilized by cell wall or extracellular carbohydrates in the root (Sanità Di Toppi and Gabbrielli, 1999). Once Cd enters the cytosol, metal ions activate the synthesis of sulfur-containing ligands such as phytochelatins or metallothioneins (DalCorso et al., 2008) and these ligand-metal complexes are stored in special cellular compartments.

A previous study in Arabidopsis detected important genes encoding enzymes and proteins involved in sulfur assimilation, GSH metabolism, indole-3-acedic acid (IAA) and jasmonic acid (JA) biosynthesis activated by lead treatment (Liu et al., 2009). In rice, Lin et al. (2013) revealed that small heat shock proteins, sulfate assimilation and glutathione-S-transferase are specifically upregulated with Cd stress. Fusco et al. (2005) identified transcription factors putatively responsive to heavy metal stress indicating the complexity of the response of plants to Cd stress. It was showed that the *TaHsfA4a* gene plays a direct role in a Cd-induced transcriptional response in wheat and rice. Besides, metallothioneins are regulated by this heat-shock transcription factor under Cd exposure (Shim et al., 2009).

In this study, we analyzed genes and regulatory pathways related to the response of Cd stress in barley. Firstly, we measured the Cd concentration in both shoot and root tissue. Second, we monitored the cell cycle to examine the effect of Cd on cell progression through individual cell phases. Third, barley root and shoot tissues were subjected to transcriptome profiling. We identified several Cd responsive genes based on differential gene expression analysis between Cd-treated material and the control. Finally, we characterized the upregulated genes encoding Cd^2+^ transmembrane transporters PLANT CADMIUM RESITANCE 2 (HvPCR2) by heterologous overexpression in yeast.

## Materials and Methods

### Plant Material and Growth Conditions

Seeds used in the experiments were kindly provided by Genebank Gatersleben of the Leibniz Institute of Plant Genetics and Crop Plant Research. Barley seeds (*Hordeum vulgare* cv. Morex) were germinated at 20°C in the dark on filter paper moistened with deionized water. After 48 h the primary root length was measured for seeds that were then transferred to polypropylene pots containing 1L of test medium (changed every 24 h to maintain composition). Test media were aerated throughout the exposure time. Relative humidity was 50%, and day/night temperatures were 20/16°C during 16/8 h photoperiod.

### Preparation of Test Solutions and Cd Accumulation Analysis

The basal nutrient solution composition was KH_2_PO_4_, 0.4 mM; K_2_SO_4_, 0.4 mM; MgSO_4_ × 7H_2_O, 0.6 mM; NH_4_NO_3_, 1 mM; Ca(NO_3_)_2_ × 4H_2_O, 2 mM; FeNaEDTA, 75 μM; MnCl_2_ × 4H_2_O, 7 μM; ZnCl_2_, 3 μM; CuSO_4_ × 5H_2_O, 0.8 μM; H_3_BO_3_, 1.6 μM, and Na_2_MoO_4_ × 2H_2_O, 0.83 μM. The pH was adjusted with 1 M KOH to 5.5–6.0 (Podar, 2013). Test media, except control, were prepared by adding different volumes of stock solutions of CdCl_2_ to adjust the concentration.

To determine Cd accumulation in barley cv. Morex, plants were cultivated in control medium and medium supplemented with 80 μM cadmium, a previously determined effective concentration (EC_50_) causing a 50% inhibitory effect for root growth (Kintlová et al., 2017). Samples were collected and root length measured before and after 5 days of treatment. Both root and shoot tissue samples were washed first in 0.5 M EDTA to remove unabsorbed cadmium, then washed in distilled water. They were further dried in a hot air oven (50°C, 72 h). The cadmium concentration was then measured by atomic absorption spectrometry (AAS).

### Ethynyl-2′Deoxyuridine Cell Proliferation Assay

#### Preparation of Plant Material

The experimental design consisted of three types of treatment to determine whether the damage of root tip cells caused by cadmium treatment is reversible or irreversible at set conditions. We examined control plants, cadmium-treated plants and washed cadmium-treated plants (designated in this study as Treatment 1–3). The experiment was repeated twice.

For all types of treatment, barley seeds were germinated (*Hordeum vulgare* cv. Morex) at 20°C in the dark on filter paper moistened with deionized water. After 48 h, 20y seeds were transferred to polypropylene pots containing 1L of test medium (changed every 24 h). Test media were aerated throughout the exposure time. Relative humidity was 50%, and day/night temperatures were 20/16°C during 16/8 h photoperiod. After 5 days, the plants in Treatment 3 were rinsed in deionized water 3 times and transferred to basal nutrition solution for 3 days, whereas Treatment 1 and 2 plants were handled immediately.

#### Plant Nuclei Extraction and 5-Ethynyl-2′-Deoxyuridine Labeling

The plant nuclei extraction protocol and EdU labeling were performed on the same day for all types of treatment. Click-iT EdU Alexa Fluor 488 Flow Cytometry Assay Kit (Invitrogen, Carlsbad, CA, USA) was used for the cell proliferation assay. Twenty barley plants of each treatment were rinsed 3 times in deionized water and placed into Petri dish filled with 50 ml of fresh deionized water. 5-ethynyl-2′-deoxyuridine (EdU) was added to a final concentration of 9 μM and incubated for 2 h.

Roots with a length of about 1 cm were trimmed and incubated 20 min in 2% (v/v) formaldehyde fixative on ice. The roots were then washed in Tris buffer three times for 5 min on ice. About 70 meristem root tips per one treatment were excised and then hmogenized in 1 ml LB01 lysis buffer using a Polytron PT1200 homogenizer at 15,000 rpm for 13 s. The crude homogenate was filtered through a nylon mesh filter (50 μm pore size) into a new tube. The tubes were spun down at 400 g and 4°C for 10 min and the supernatant removed. The Click-iT reaction cocktail was prepared using 438 μl PBS, 10 μl CuSO_4_, 2.5 μl Fluorescent dye azide and 50 μl Reaction Buffer Additive for each sample. 500 μl of reaction cocktail was applied to the pelet, mixed well and incubated for 30 min in the dark at room temperature. The solution was spun down again and the pelet was resuspended in 500 μl LB01 buffer. The suspension was filtered through a nylon mesh filter (25 μm pore size) and stained with DAPI (2 μg/ml final concentration).

#### Cell Cycle Analysis Using Flow Cytometry

All flow cytometric analyses were performed on FACSAria SORP flow cytometer (BD Biosciences, Santa Clara, USA) equipped with blue (488 nm, 100 mW) and UV (355 nm, 100 mW) lasers and optical filters for FITC (530/30 band-pass) and DAPI (450/50 band-pass) fluorescence detection. At least 10,000 nuclei were recorded for each sample. Data were displayed on Alexa Fluor 488 vs. DAPI dot plots. To make statistical evaluation of the effect of cadmium treatment on cell proliferation, the region around cycling and non-cycling populations were drawn, respectively. The ratio of cycling/non-cycling was calculated for each sample and the mean and the standard deviation were calculated from three repetitions.

### Transcriptome Analysis

For transcriptome analysis we used the dataset available in NCBI SRA (sequence read archive) under accession numbers SRR5452097 to SRR5452108. These data were obtained from the barley plants grown in media supplemented with cadmium concentration that corresponds to established EC_50_ values as well as control plants for each treatment (Kintlová et al., 2017). Sequenced Read quantification of each dataset was performed using Salmon (Patro et al., 2017) and 63,658 *H. vulgare* CDS (Mascher, 2019) and 3,349,186 repeats (IBSC, 2016). Differential expression analysis was then conducted using R and the package DESeq2 (Love et al., 2014). Gene ontologies were retrieved from the available barley gene annotation (Mascher, 2019) and were further analyzed with the DE genes. Bioinformatic analysis of RNA-seq data was carried out in the computing and storage facilities MetaCentrum infrastructure.

### qRT-PCR Validation of Selected Differentially Expressed Transcripts

The mRNA levels for selected differentially expressed transcripts was validated by qRT-PCR. As representatives, most up- and down-regulated transcripts from both root and shoot tissues were selected for analysis (Supplementary Table 3). Plant material was grown and treated as described above. The RNA from control and Cd-treated plants (80 μM) was isolated in triplicate by NucleoSpin RNA Plant kit (Macherey-Nagel, Germany) and transcribed to cDNA by Transcriptor high fidelity cDNA synthesis kit (Roche). The qRT-PCR reactions were performed on a Light Cycler 96 real-time PCR system (Roche, USA) using the 2x SYBR Master Mix (Top-Bio, Czech Republic). Relative expression of shoot and root transcripts was assessed using CFX Manager™ Software (Bio-Rad, Hercules, California, USA).

### *HvPCR2* Sequence Analysis

Amino acid identity for each *HvPCR2* gene and previously described *Arabidopsis PCR* genes (Song et al., 2004, 2010) was determined using alignment algorithm MAFFT v7.450 (Katoh and Standley, 2013). The genomic sequence of *Hordeum vulgare* (IBSC_v2), *Oryza sativa Indica* group (ASM465v1) *Zea mays* (B73_RefGen_v4) and *Musa acuminata* (ASM31385v1) were used to analyze genomic loci containing *PCR2* homologs, data were retrieved and visualized using EnsemblPlants website (Howe et al., 2020).

### Heterologous Expression in Cd-Sensitive Yeast Mutant

To test the function of *HvPCR2* genes, all five identified copies were expressed in *Saccharomyces cerevisiae* cadmium hypersensitive mutant strain DTY168 (*MAT*α, *his6, leu2-3*−*112, ura3-52, ycf1::hisG*) lacking the *yeast cadmium factor 1* gene (Szczypka et al., 1994). *HvPCR2.1-5* coding sequences were amplified from Morex cDNA (Supplementary Table 4) and cloned into p426 under the control of yeast GDP (glyceraldehyde-3-phosphate dehydrogenase) promoter (Mumberg et al., 1995). Vectors containing *HvPCR2* fragments and empty vector p426 were introduced into the DTY168 strain by lithium acetate. Yeast transformants were grown overnight, OD600 was adjusted to 0.5, four 10-fold dilutions were made, and each culture was spotted on SD-Ura plates and SD-Ura plates supplemented with 50 μM CdCl_2_. Plates were incubated for 5–6 days at 28°C.

## Results

### Barley Growth and Cell Proliferation Inhibition by Cd Stress

**Figure 1:**
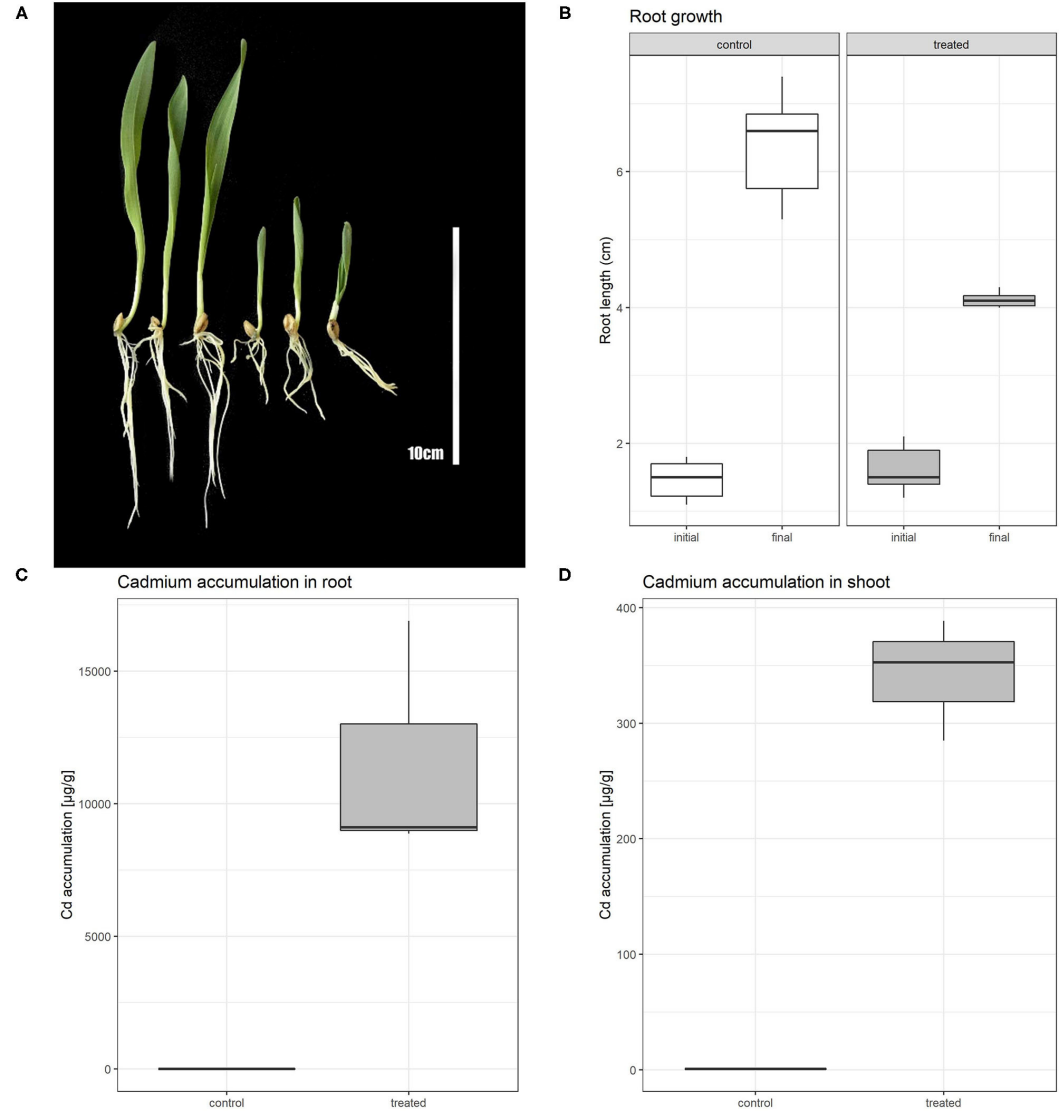
Phenotypic comparison of control and Cd treated plants **(A)**; left section represents control plants, right section Cd treated plants. Boxplot showing root length of barley plants at the beginning of experiment (initial) and after 5 days of Cd or control treatment (final) **(B)**. Boxplot of cadmium accumulation for both control and treated plants in root **(C)** and shoot **(D)**.

The effect of 80 μM Cd on barley after a 5-day treatment comprised of root shortening and overall growth retardation (Figure 1A). Root growth of Cd-treated plants was inhibited by ~50% in comparison with non-treated plants (Figure 1B; Supplementary Table 1). Further, we measured the Cd content in control and treated plants in order to obtain information about the distribution of Cd in barley plants in both root and shoot tissues. The Cd content in roots was in control conditions approximately double of the Cd concentration in shoots. Cd-treated plants accumulated roughly 6,600 times more Cd in roots than non-treated plants. This result shows a strong accumulation of Cd in the root and restriction of Cd translocation to the aerial tissues in high Cd stress (Figures 1C,D; Supplementary Table 2).

**Figure 2:**
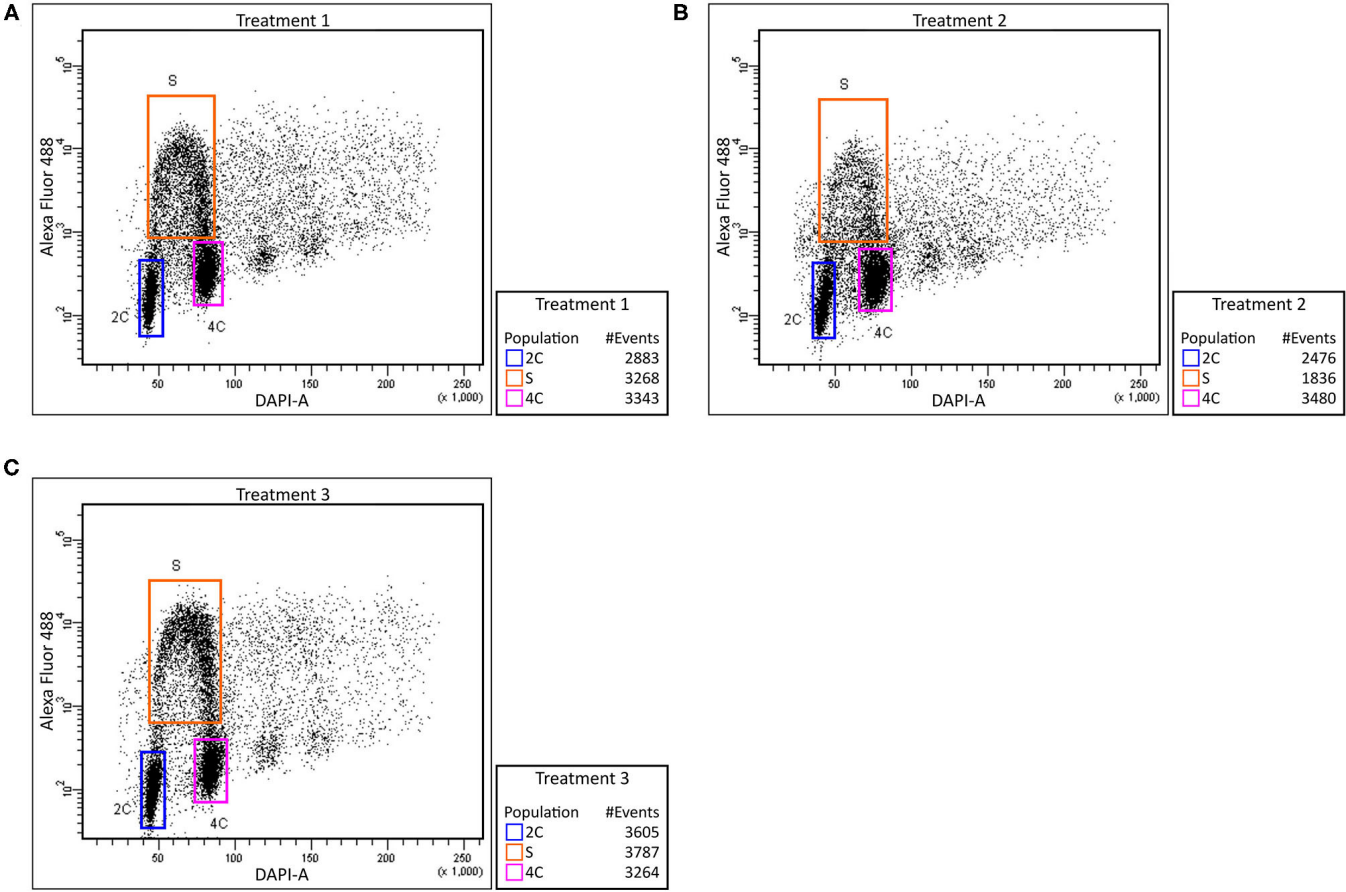
Effect of cadmium on root cell cycle. Barley root tip cells were labeled with EdU and analyzed using flow cytometry. Control plants (no added Cd)–Treatment 1 **(A)**, plants treated with Cd–Treatment 2 **(B)**, and plants washed and grown in control media after Cd exposure–Treatment 3 **(C)**. Relative DNA content was measured using DAPI (X-axis) and extent of proliferating nuclei was measured by means of EdU incorporation based on Alexa Fluor 488 fluorescence (Y-axis). Regions of replicating (orange) and non-replicating (blue and purple) nuclei are indicated.

The labeled thymidine analog (EdU) was used to monitor cell cycle abnormalities of Cd treated plants and to determine the Cd effect on root growth at the cellular level. In control plants, we observed an approximately equal number of cells before, during and after the S phase (Figure 2A). Cd-treated roots contained a significantly lower number of S phase cells (Figure 2B). Such a decrease may suggest that the cells entering the S phase are the most susceptible to Cd treatment. After removing the Cd by washing the roots, the number of cells in each state went back to the level similar to the non-treated control (Figure 2C). These findings indicate that the major reduction in root growth is caused by the suppression of the cell division process namely in G1/S cells.

### Identification of Transcripts Deregulated by Cd

**Figure 3:**
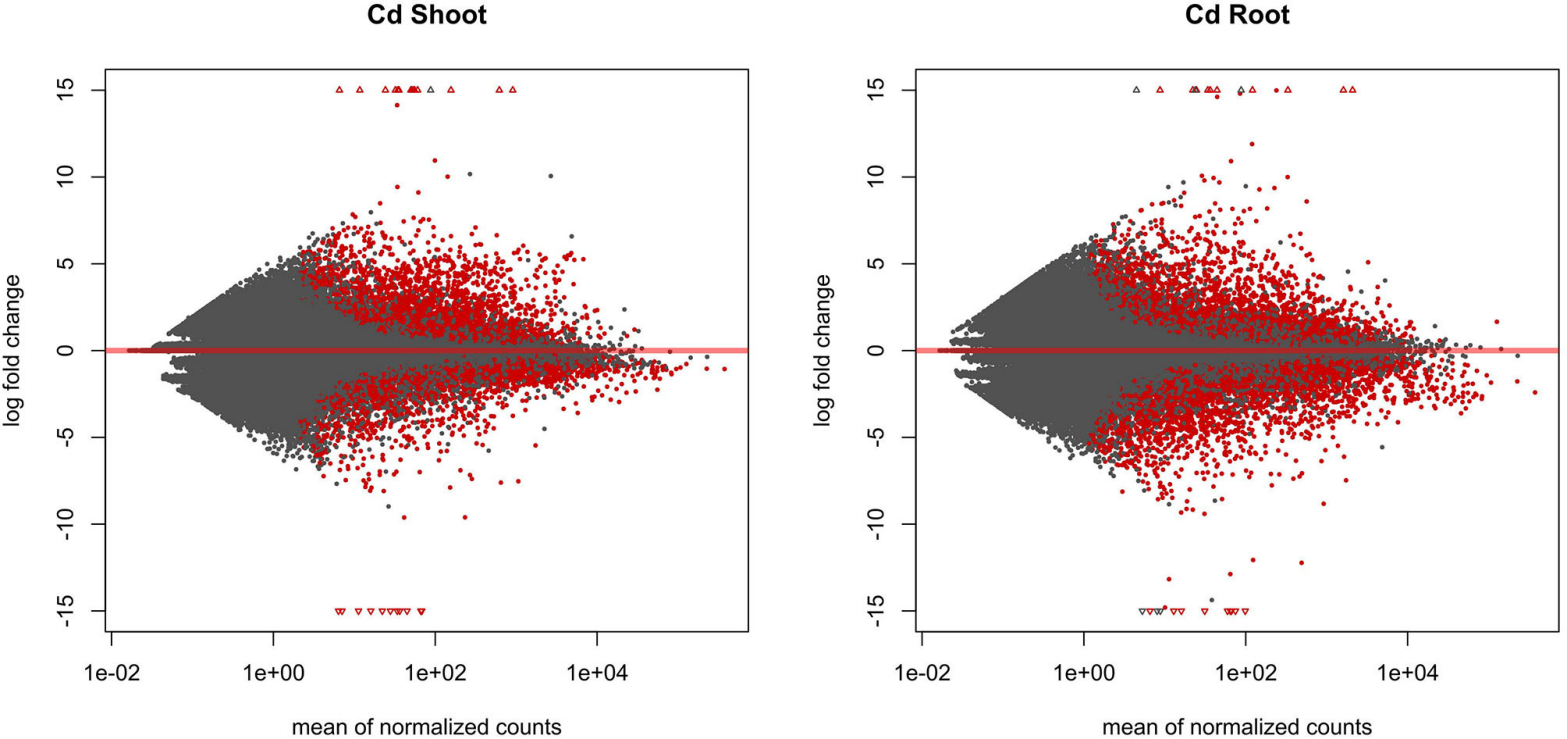
MA plot displaying the log-fold change (logFC) for each gene against the mean of normalized counts in shoot and root tissue. Differentially expressed genes with a *p*-value below 0.05 are highlighted in red.

**Figure 4:**
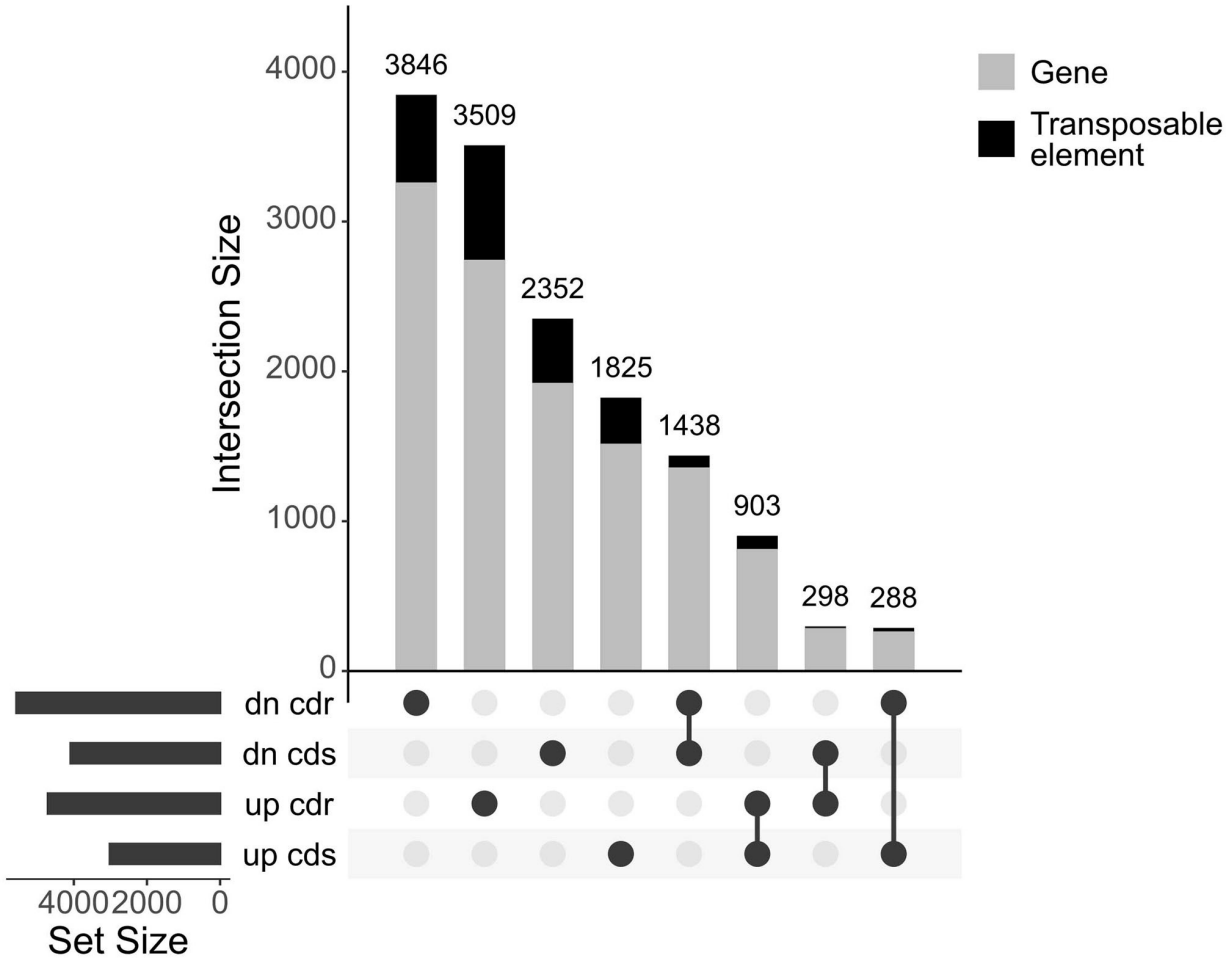
UpSet plot of the differentially expressed transcripts under cadmium treatment comparing response in root and shoot. Transcripts with a *p*-value below 0.05 were retained. Count of transcripts are displayed over the bars for each relationship of up and down (dn)-regulated transcripts from both root (cdr) and shoot (cds). The bars are colored according to the proportion of transcripts coming from both genes and transposable elements in gray and black, respectively.

The entire dataset subjected to differential expression analysis contains 20.7 million of non-treated and 24.3 million of cadmium treated RNA reads (Kintlová et al., 2017). The analysis was performed using the DESeq2 package from Bioconductor (Love et al., 2014). We controlled the clustering of both control and cadmium replicates using multidimensional scaling for each tissue (Supplementary Figure 1). We identified 14,459 differentially expressed (DE) transcribed sequences in both root and shoot tissues using a *p*-value threshold of 0.05 (Figure 3). The overall count of DE after cadmium treatment is 14,459 (Figure 4; Supplementary Tables 6, 7) with 2,927 transcripts that have a significant change in their expression in both tissues while 7,355 were differentially expressed only in the root and 4,177 in the shoot. Separating the set of DE transcripts between up- and down-regulated reveals that most of the transcripts in the root and shoot are down-regulated (3,509 up vs. 3,846 down in root and 1,825 up vs. 2,352 down in shoot). Differentially expressed transcripts present in both tissues follow the same pattern of expression change with more down-regulated transcripts than up-regulated ones. From the 2,927 DE transcripts in both tissues, 903 are up-regulated, and 1,438 are down-regulated in roots and shoots. Nevertheless, 288 transcripts are down-regulated in roots while up-regulated in shoots, and 298 transcripts are up-regulated in roots while down-regulated in shoots (Figure 4).

Analysis of the transcripts related to repeated elements revealed that 2,285 elements (from total 14,459 DE transcribed sequences) are differentially expressed (Supplementary Figure 2). 59% are specifically differentially expressed in the root while 32% in the shoot. About 75% of the total of DE transposable elements are long terminal repeats (LTR). Transposable element related transcripts are more expressed in roots than shoots, similar to the genes-related ones, but unlike gene transcripts, they are more often up-regulated. Most of the expressed LTR belong to an unknown subfamily; otherwise, DE LTR *Gypsy* are more abundant than LTR *Copia*, followed by CACTA, nonLTR/LINE, and MITE elements (Supplementary Figure 3; Supplementary Table 6).

**Figure 5:**
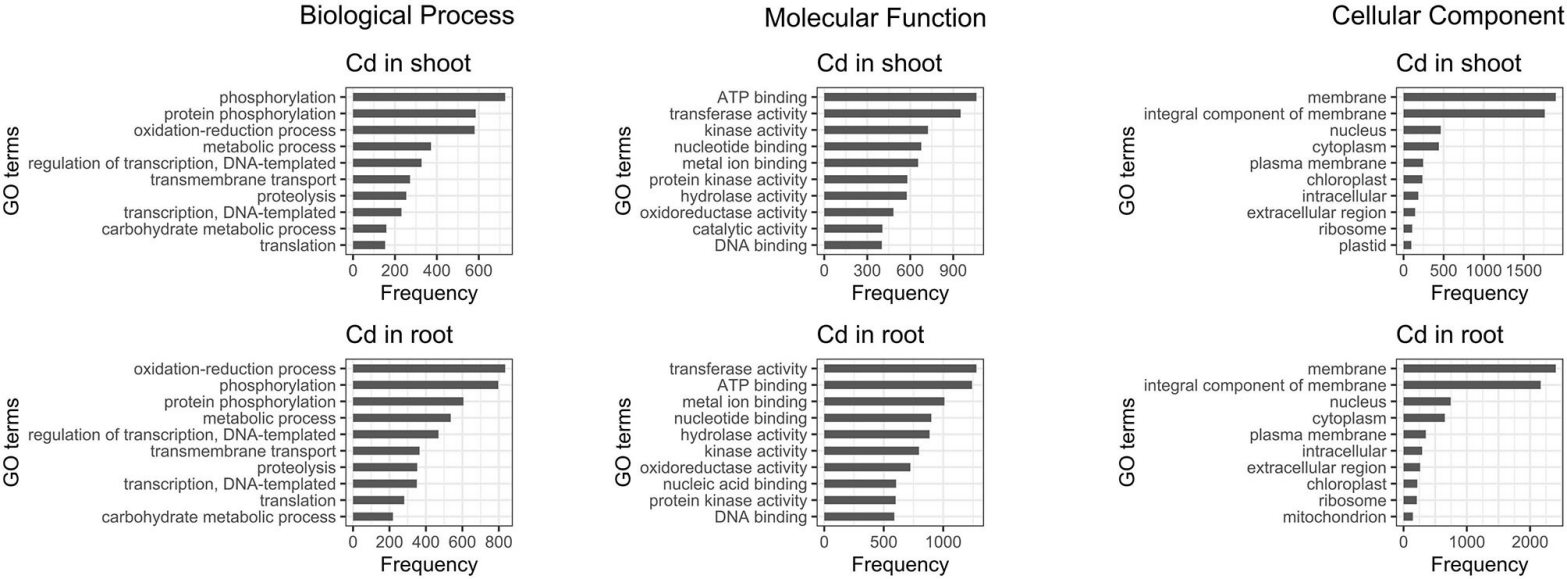
Ten most abundant gene ontology of differentially expressed genes present in roots and shoots. The 10 most abundant biological process, molecular function, and cellular component gene ontology (GO) are displayed for both root and shoot tissues.

The 10 most abundant gene ontology (GO) annotations for both roots and shoots were assessed for DE transcripts. Comparison of the results reveals that regardless of the tissue, most of the genes influenced by cadmium are: involved in oxidation-reduction processes and phosphorylation, have mainly ATP binding or transferase activity functions and are part of the membrane. Moreover, the genes involved in protein phosphorylation and protein kinase activity are also influenced, but the majority of them are up-regulated while the genes involved in catalytic activity are mainly down-regulated (Figure 5).

### qRT-PCR Validation

In order to validate RNA-seq results, we selected a total of six transcripts for which we designed qPCR primers (Supplementary Table 3). *HORVU.MOREX.r2.7HG0558200* is up-regulated in shoots, *HORVU.MOREX.r2.6HG0456470* is down-regulated in shoots and *HORVU.MOREX.r2.1HG0006620* is not differentially expressed in shoots. *HORVU.MOREX.r2.2HG0162330* is up-regulated in roots, *HORVU.MOREX.r2.7HG0538720* is down-regulated in roots and *HORVU.MOREX.r2.7HG0561140* is not differentially expressed in roots (Supplementary Figure 4). The qPCR results correspond to the mRNA level identified by differential expression analysis for each of the six candidates (Supplementary Figure 5).

### *HvPCR2* Genes Are Upregulated by Cd and Restore Tolerance in Cd Sensitive Yeast Mutant

**Figure 6:**
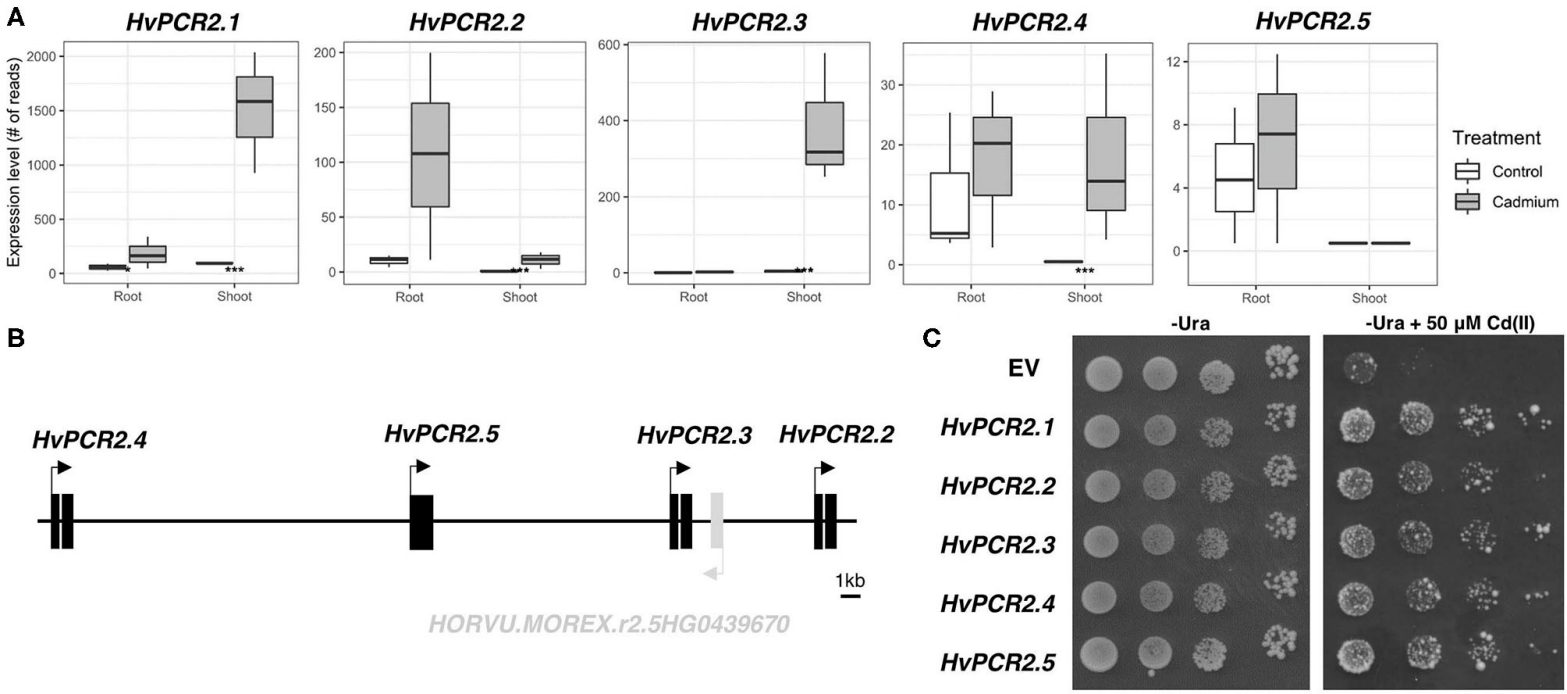
Expression, gene cluster structure, and functional analyses of *HvPCR2*. Transcription profiles of all *HvPCR2* genes in control and after Cd exposure in root and shoot tissue **(A)**. Box plot showing the expression level of a gene as count of reads for both root and shoot tissues in control and cadmium treated conditions. The difference between control and treatment was statistically tested and the adjusted *P*-value < 0.05 is displayed as an asterisk (*), similarly *P*-value < 0.005 is displayed as (***). Schematic diagram of the ~35 kb long genomic region of barley chromosome 5H containing *HvPCR2.2-5* genes in tandem array **(B)**. Expression of *HvPCR2* genes restore the Cd tolerance in yeast mutant DTY168 (Δ*ycf1*) **(C)**. Growth of DTY168 yeast cultures transformed with empty vector (EV) or constructs containing *HvPCR2* genes on solid media with and without addition of Cd(II).

Among the most Cd upregulated transcripts, we identified homologs of *PLANT CADMIUM RESISTANCE 2* (*PCR2*) gene. Predicted protein sequences show the highest similarity with AtPCR2 protein (Supplementary Table 5), therefore we have designated these genes *HvPCR2.1* (*HORVU.MOREX.r2.1HG0071220), HvPCR2.2* (*HORVU.MOREX.r2.5HG0439660*), *HvPCR2.3* (*HORVU.MOREX.r2.5HG0439680*), and *HvPCR2.4* (*HORVU.MOREX.r2.5HG0439700*). While the mRNA level of *HvPCR2.1* is significantly upregulated by Cd in both root and shoot tissue, *HvPCR2.2-4* are upregulated only in the shoot (Figure 6A). Surprisingly, *HvPCR2.2-4* were found to be located in the same locus on barley chromosome 5H (Figure 6B). The fifth copy of *HvPCR2* gene (*HORVU.MOREX.r2.5HG0439690*; denominated *HvPCR2.5*) is also physically present in this ~35 kb spanning region as well (Figure 6B). However, *HvPCR2.5* does not exhibit significant upregulation upon Cd treatment. Similar tandem arrangement of *PCR2* homologs have been found in rice (~30 kb locus on chromosome 3 containing six *PCR2* homologs), corn (~90 kb locus on chromosome 5 containing three *PCR2* homologs) and banana (~30 kb locus on chromosome 3 containing three *PCR2* homologs) (Supplementary Figure 23).

To elucidate the function of *HvPCR2* genes in metal tolerance, we expressed them in the cadmium sensitive yeast strain DTY168 (Δ*ycf1*), which showed restricted growth at increased Cd concentrations. Growth of the DTY168 harboring empty vector p426 GDP was strongly inhibited by 50 μM CdCl_2_, whereas the same yeast mutant strain expressing either of the *HvPCR2* genes was able to restore Cd tolerance (Figure 6C).

## Discussion

Cadmium is known to inhibit the growth of wheat (Sun et al., 2005) and other important crop relatives to barley (Rizwan et al., 2016; Ling et al., 2017). RNA-seq based comparative transcriptomics have been successfully used to uncover the mechanisms of plant stress response (Tombuloglu et al., 2015; Haak et al., 2017). In this study, we analyzed the transcriptome profiles of barley plants affected by elevated cadmium concentrations. We have selected *Hordeum vulgare* cv. Morex because of the availability of its genome and reference transcriptome sequence (Mascher, 2019).

### Cd Concentration in Barley Plants and Cd Effect on Cell Proliferation

To provide quantitative information about the presence of cadmium in barley plants, we measured the Cd concentration in roots and shoots separately (Figures 1C,D). We found that under cadmium treatment roots and shoots were able to accumulate a high concentration of this element. While Cd-treated shoots accumulate up to 300 times more Cd than in the non-treated condition, roots accumulated more than 6,600 times of this element. This difference indicates the limitations in the transport of cadmium in the aerial part of the plant.

Monitoring the cell cycle by EdU labeling revealed a lower number of S phase cells in Cd-treated roots. Such a decrease may suggest that the cells before G1/S transition are the most susceptible to Cd treatment. The recovery of the number of cells after removing the Cd by washing the root indicates that the major reduction in root growth is caused by the suppression of the cell division process namely in G1 phase cells (Figure 2). While the negative influence of Cd toxicity on cell proliferation is well-known in barley and wheat (Zhang and Yang, 1994; Paradiso et al., 2008), this phenomenon is not yet fully understood. Presumably, a Cd-induced ROS accumulation may cause an oxidative posttranslational modification of cyclin D and A-type cyclin-dependent kinase, thereby reducing the functionality of essential G1/S transition regulators (Pena et al., 2012). Similar reductions in the number of S phase cells have been reported in Cd-treated *Zea mays* (Bertels et al., 2020), *Sorghum bicolor* (Zhan et al., 2017), and *Vicia faba* (Zabka et al., 2021) as well as in Al-treated barley (Jaskowiak et al., 2018). Although Cd is also known to cause abnormal mitosis and aberrant chromosomes in barley (Shi et al., 2016), our data suggest a substantial effect of Cd toxicity at the level of G1-to-S-phase transition.

### Differentially Expressed Transcripts

In our study, we identified over 14,400 transcripts differentially expressed in Cd treated plants, with about half of them being down-regulated. The total number of DE transcripts were lower in shoots (7,104) than in roots (10,282), consistent with the Cd concentration level detected in both tissues (Figures 1C,D). Previous transcriptomic studies reporting the effect of cadmium on barley focused on Cd tolerance, rather than response to Cd excess (Cao et al., 2014; Derakhshani et al., 2020). As the general objective of our study was to describe the overall response to cadmium in barley, we identified higher number of differentially expressed transcripts.

First, we analyzed the repetitive fraction of the barley genome at the transcriptional level to quantify the impact of cadmium on the activity of individual elements. Abiotic stress is frequently accompanied by the transcriptional and transpositional activation of transposable elements (TEs) (Grandbastien, 1998). Moreover, cadmium related stress leads to the establishment of a new balance of expressed/repressed chromatin (Greco et al., 2012). We found that the vast majority of transcriptionally affected repeats represent LTR retrotransposons (*Copia, Gypsy* and unknown families) and CACTA, nonLTR/LINE and MITE elements (Supplementary Figure 3). Transcriptional response to cadmium treatment is more evident in root tissue (Supplementary Figures 2, 3) including both upregulation and downregulation of individual TEs (Supplementary Table 6).

### Stress Response and ROS Metabolism

DE transcripts are mostly connected with oxidation-reduction processes, protein phosphorylation, metabolic processes, regulation of transcription and transmembrane transport (Figure 5). Among them, the most upregulated transcripts match genes encoding proteins involved in ROS metabolism and oxidative stress such as tau class glutathione-S-transferase (GST, Supplementary Figures 6, 7), purple acid phosphatase (PAP, Supplementary Figure 8), multidrug resistance-associated protein 3 (MRP3, Supplementary Figure 9), and cytochrome P450 enzymes (P450, Supplementary Figures 10, 11). All of these are also known to play a role in the detoxification of xenobiotics or abiotic stress signaling (Bovet et al., 2003; Schenk et al., 2013; Kumar and Trivedi, 2018; Pandian et al., 2020). Cadmium is known to induce oxidative stress in plants (Grobelak et al., 2019); therefore, the enhancement of ROS scavenging mechanisms represents a straightforward reaction to elevated Cd concentration. In particular, increased expression of GST and P450 enzymes in response to Cd stress has been previously described in rice (Ogawa et al., 2009) and the simultaneous overexpression of both enzymes in *Medicago truncatula* led to improved tolerance to mercury contamination (Zhang et al., 2013). In addition, four genes encoding homologs to putative chromatin remodeler OXIDATIVE STRESS 3 (OX3) were upregulated (Supplementary Figure 12). Although the molecular mechanism of OX3 action is not completely understood, this enzyme provides tolerance to Cd and oxidative stress in *Arabidopsis* and fission yeast (Blanvillain et al., 2009).

### Cell Wall Formation and Maintenance

Other coding genes showing significant upregulation after Cd treatment, include those involved in cell wall formation and modification. Transcripts coding for lignin biosynthesis components, namely phenylalanine ammonia lyase (PAL; Supplementary Figure 13), shikimate O-hydroxycinnamoyltransferase (HCT; Supplementary Figure 14), cinnamoyl-CoA reductase (CCR; Supplementary Figure 15), cinnamyl alcohol dehydrogenase (CAD; Supplementary Figure 16), laccase 7 (LAC7; Supplementary Figure 17), and peroxidase (POD; Supplementary Figure 18) have been highly upregulated in Cd response. PAL is the key enzyme of the lignin biosynthesis acting at the beginning of this metabolic pathway. Accordingly, the *Arabidopsis pal1 pal2 pal3 pal4* quadruple mutant showed approximately four times decreased lignin content in its cell wall compared with the wild-type (Huang et al., 2010). PAL activity or expression is known to be induced by Cd stress in various plant species including pea (Głowacka et al., 2019), *Medicago sativa* (Gutsch et al., 2019), *Salix matsudana* (Yang et al., 2015), rice (Hsu and Kao, 2005), and wheat (Shakirova et al., 2016). HCT, CCR and CAD catalyze the synthesis of monolignols, which are then polymerized into lignin by LAC and POD enzymes (Liu et al., 2018). As the lignification is improving the cell wall impermeability and lignin itself binds cadmium (Parrotta et al., 2015), the enhanced expression of these enzymes is likely to result in a lower Cd uptake and a higher Cd sequestration capacity. Notably, altered lignin biosynthesis was proposed to contribute to cadmium tolerance in Zn/Cd hyperaccumulator *Noccaea caerulescens* (van de Mortel et al., 2006, 2008). Other highly Cd responsive transcripts encode homologs of wound induced protein 1 (*HORVU.MOREX.r2.1HG0053620;* Supplementary Figure 19) and Leucine-rich repeat receptor-like protein kinase (*HORVU.MOREX.r2.2HG0164280*, Supplementary Figure 19), both of which have been reported to function in cell wall integrity sensing and responding to biotic and abiotic stress (Logemann and Schell, 1989; Van der Does et al., 2017).

### Metal Transport

Metal ion transporters play significant role in heavy metal homeostasis and detoxification (Jain et al., 2018). In our dataset, five deregulated transcripts have been identified to encode the members of the ZRT/IRT-like protein (ZIP) family (Supplementary Figure 20). ZIP transmembrane proteins are known to mediate cellular uptake, intracellular trafficking and detoxification of heavy metal cations such as Zn, Fe, Cd, Cu, Mn, Co, and Ni (Ajeesh Krishna et al., 2020). The role of selected ZIP transporters has been studied extensively in monocots (Li et al., 2013; Evens et al., 2017; Zheng et al., 2018) including barley (Pedas et al., 2009; Tiong et al., 2015). Interestingly, QTL mapping in barley identified two ZIP transcripts among the candidate genes for Cd tolerance (Derakhshani et al., 2020). In our dataset, we identified three previously studied ZIP transporters–*HvZIP1* (*HORVU.MOREX.r2.3HG0273580*), *HvZIP3* (*HORVU.MOREX.r2.2HG0158440*), and *HvZIP6* (*HORVU.MOREX.r2.1HG0020460*). *HvZIP1* was found to be upregulated by Cd in both tissues, but the effect is much stronger in roots (Supplementary Figure 20). An identical expression pattern has been found for *OsZIP1*, a rice ortholog for *HvZIP1*, that functions as a heavy metal efflux transporter maintaining detoxification of Zn, Cu and Cd in rice (Liu et al., 2019). Upon cadmium exposure, the *HvZIP3* shows upregulation in roots but downregulation in shoots (Supplementary Figure 20). The transgenic barley plants with reduced *HvZIP3* expression showed a significant increase in grain Cd accumulation (Sun et al., 2015). Further, a co-expression of rice *HvZIP3* homolog (*OsZIP3*) with rice heavy metal transporter genes *HEAVY METAL ATPASE 2* (*OsHMA2*) and *LOW-AFFINITY CATION TRANSPORTER1* (*OsLCT1*) resulted in a decrease in Cd accumulation and enhanced Cd tolerance (Tian et al., 2019). *HvZIP6* shows strong upregulation in shoots only (Supplementary Figure 20). So far, *HvZIP6* was studied only in relation to Zn homeostasis (Tiong et al., 2015) but OsZIP6 is known to transport Cd^2+^ cations (Kavitha et al., 2015), therefore HvZIP6 may take part in shoot Cd detoxification. Two previously unknown barley ZIP transporters encoded by *HORVU.MOREX.r2.2HG0098160* and *HORVU.MOREX.r2.7HG0603680*, show downregulation after Cd treatment (Supplementary Figure 20), which may suggest these could mediate Cd uptake.

Another group of deregulated genes encoding cation transmembrane transporters are members of the *NATURAL RESISTANCE ASSOCIATED MACROPHAGE PROTEIN* (*NRAMP*) family. In Cd-treated roots, *HvNRAMP5* (*HORVU.MOREX.r2.4HG0337880*) exhibited several-fold downregulation (Supplementary Figure 21), which is consistent with the role of HvNRAMP5 as a Cd uptake transporter (Astolfi et al., 2014; Wu et al., 2016). Conversely, the *HvNRAMP5* mRNA level slightly increased after Cd excess in shoot, which may imply involvement of this gene in shoot Cd distribution in stress condition. The *HvNRAMP2* (*HORVU.MOREX.r2.4HG0331160*) were downregulated in shoots (Supplementary Figure 21). As the genome-wide association mapping study has proposed *OsNRAMP2* to be responsible for Cd accumulation (Zhao et al., 2018), the downregulation of this transcript in barley might serve as a defense against accumulation of high Cd concentrations in shoot. The two remainder transcriptionally deregulated transporters from the *NRAMP* family were *HvNRAMP1* (*HORVU.MOREX.r2.7HG0610240*; upregulated in roots) and *HvNRAMP6* (*HORVU.MOREX.r2.3HG0215920*; upregulated in shoots) (Supplementary Figure 21).

Two transcripts encoding HEAVY METAL ATPASE (HMA), a P-type ATPase cation transporter family, were identified as being responsive to Cd. *HvHMA1* (*HORVU.MOREX.r2.5HG0401460*) is expressed only in shoot tissue and no transcripts are detected after Cd treatment (Supplementary Figure 22). This pattern is similar to results published previously (Mikkelsen et al., 2012) and suggests that Cd induced downregulation of protoplast- and aleurone layer-localized HvHMA1 helps to prevent the cadmium transport into grains and protoplasts. The mRNA level of *HvHMA3* gene (*HORVU.MOREX.r2.7HG0603650*) was decreased by Cd treatment (Supplementary Figure 22). Unfortunately, the function of this *HMA3* copy has not been studied yet, so it is hard to clarify the effect of its downregulation. On the other hand, different *HvHMA3* gene (*HORVU5Hr1G094430*) has been proposed to play a crucial role in grain Cd accumulation (Wu et al., 2015; Lei et al., 2020).

Remarkably, four homologs of *PLANT CADMIUM RESISTANCE 2* (*PCR2)*, encoding a Cys-rich domain containing membrane Cd/Zn efflux transporter (Song et al., 2010), have been identified among the Cd upregulated transcripts in shoot (Figure 6A). *PCR1* and *PCR2* genes were previously studied in *Arabidopsis* and it was proposed that they play a role in Zn/Cd detoxification and distribution (Song et al., 2004, 2010). The *Arabidopsis pcr2* loss-of-function mutant is sensitive to Cd (Song et al., 2010). In rice, overexpression of *OsPCR1* and *OsPCR3* mediated Cd tolerance and lowered Cd accumulation (Wang et al., 2019). As there are no data regarding the function of *PCR2* genes in monocots, we expressed barley *HvPCR2* genes in a Cd sensitive yeast mutant. All five *HvPCR2* genes restored the Cd tolerance in yeast (Figure 6C), hence it is probable that they retain a similar function as *Arabidopsis* homologs. Moreover, we have found out that four *HvPCR2* genes are located on barley chromosome 5H in tandem arrangement (Figure 6B), probably due to local gene duplication events. A similar arrangement of multiple *PCR2* copies was found in genomic regions of rice, corn and banana (Supplementary Figure 23), suggesting that *PCR2* multiplication might be common in monocots. Duplication of heavy metal transporter genes is known to contribute to Cd tolerance in metal hyperaccumulators *Arabidopsis halleri* and *Noccaea caerulescens* (Hanikenne et al., 2008; Ó Lochlainn et al., 2011). Beside increasing gene dosage, the duplication may also allow functional divergence. It is therefore possible that the increased copy number of *PCR2* genes originated as an adaptive response to Cd toxicity and/or Zn homeostasis requirements.

## Conclusion

In summary, we investigated the impact of cadmium excess at the transcriptional level in barley. Among Cd-induced transcripts, we have identified the *HvPCR2* gene as one of the most cadmium responsive. Surprisingly, further analysis suggested local multiplication of the gene on barley chromosome 5H as well as in other monocot species. Using gene complementation in yeast mutants we confirmed the functionality of individual *HvPCR2* copies. This study may provide a valuable resource for the potential exploitation of the role of the *HvPCR2* gene complex in cadmium tolerance in plants.

## Data Availability Statement

The datasets generated for this study can be found in online repositories. The names of the repository/repositories and accession number(s) can be found below: Sequenced reads are available in NCBI SRA (sequence read archive) under accession SRR5452097 to SRR5452108 (https://www.ncbi.nlm.nih.gov/bioproject/PRJNA382490).

## Author Contributions

MK and VH conducted the research in the laboratory. Computational analysis done by NB. All authors were involved in planning the experiments and contributed to the manuscript writing.

## Conflict of Interest

The authors declare that the research was conducted in the absence of any commercial or financial relationships that could be construed as a potential conflict of interest.
