# Supplementary material for: Transcriptome Response to Cadmium Exposure in Barley (Hordeum vulgare L.)
Source: Front Plant Sci. 2021 Jul 15;12:629089. doi: 10.3389/fpls.2021.629089 (PMC8321094; doi:10.3389/fpls.2021.629089)
Supplement: Supplementary file 1 [file Data_Sheet_1.DOCX]

Supplementary Material – Figures 1-5

**
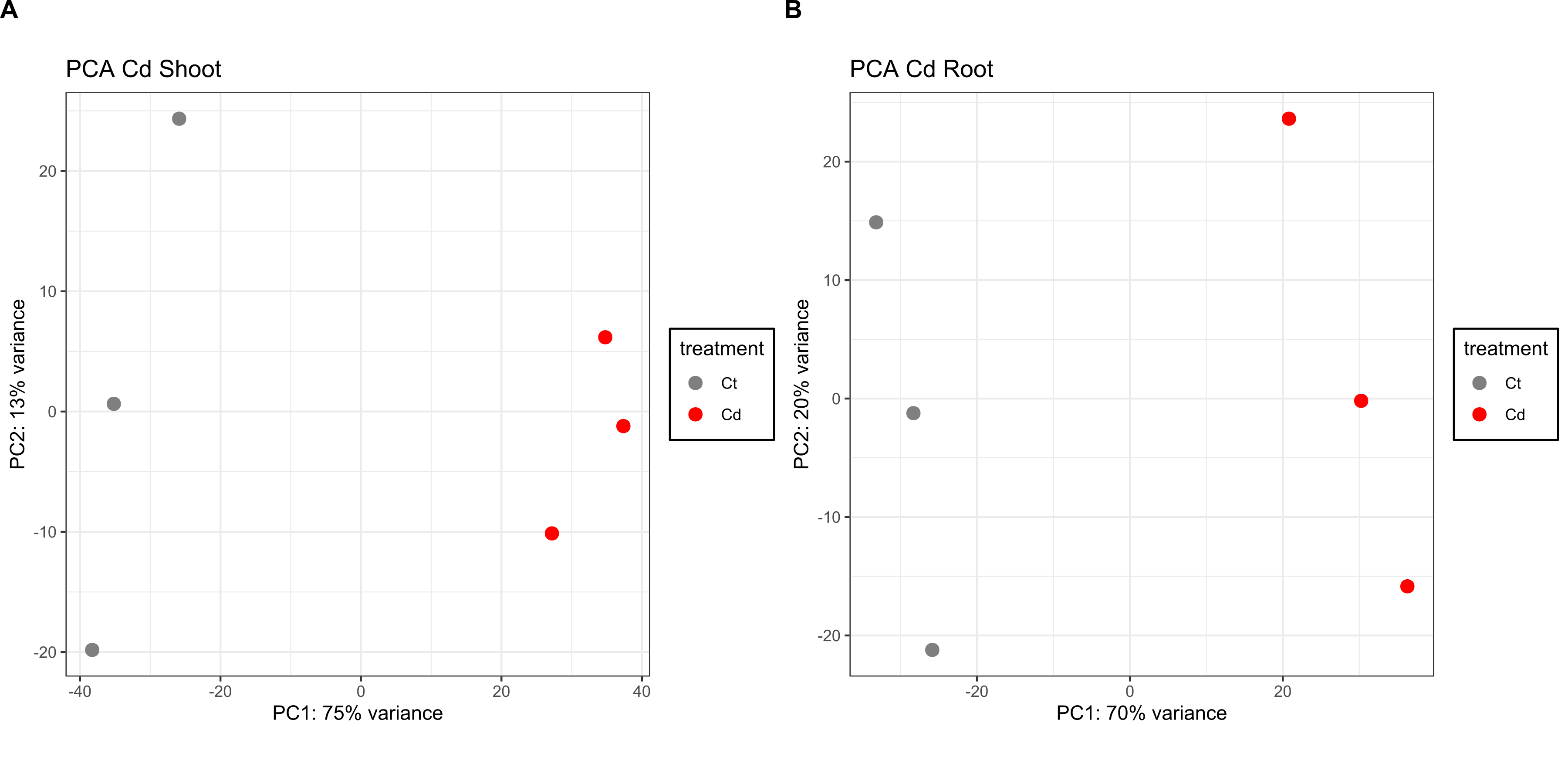
Supplementary Figure 1.** RNA-seq data plots. Principal component analysis (PCA) plots show that control sample are different than treated samples in both shoot (A) and root (B) experiment.

####
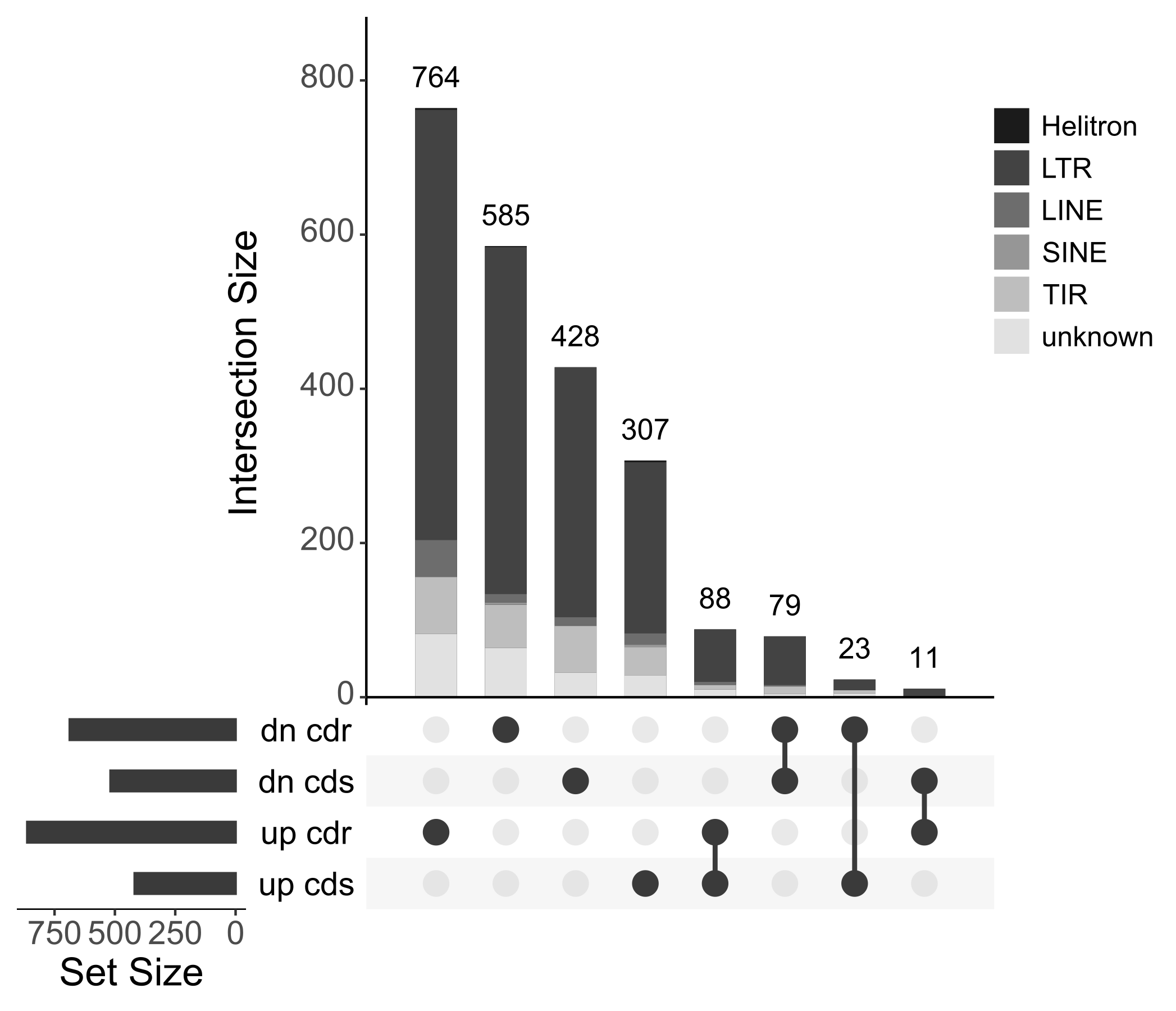
Supplementary Figure 2. UpSet plot of differentially expressed repeated elements. Bar are colored according to the transposable element order.


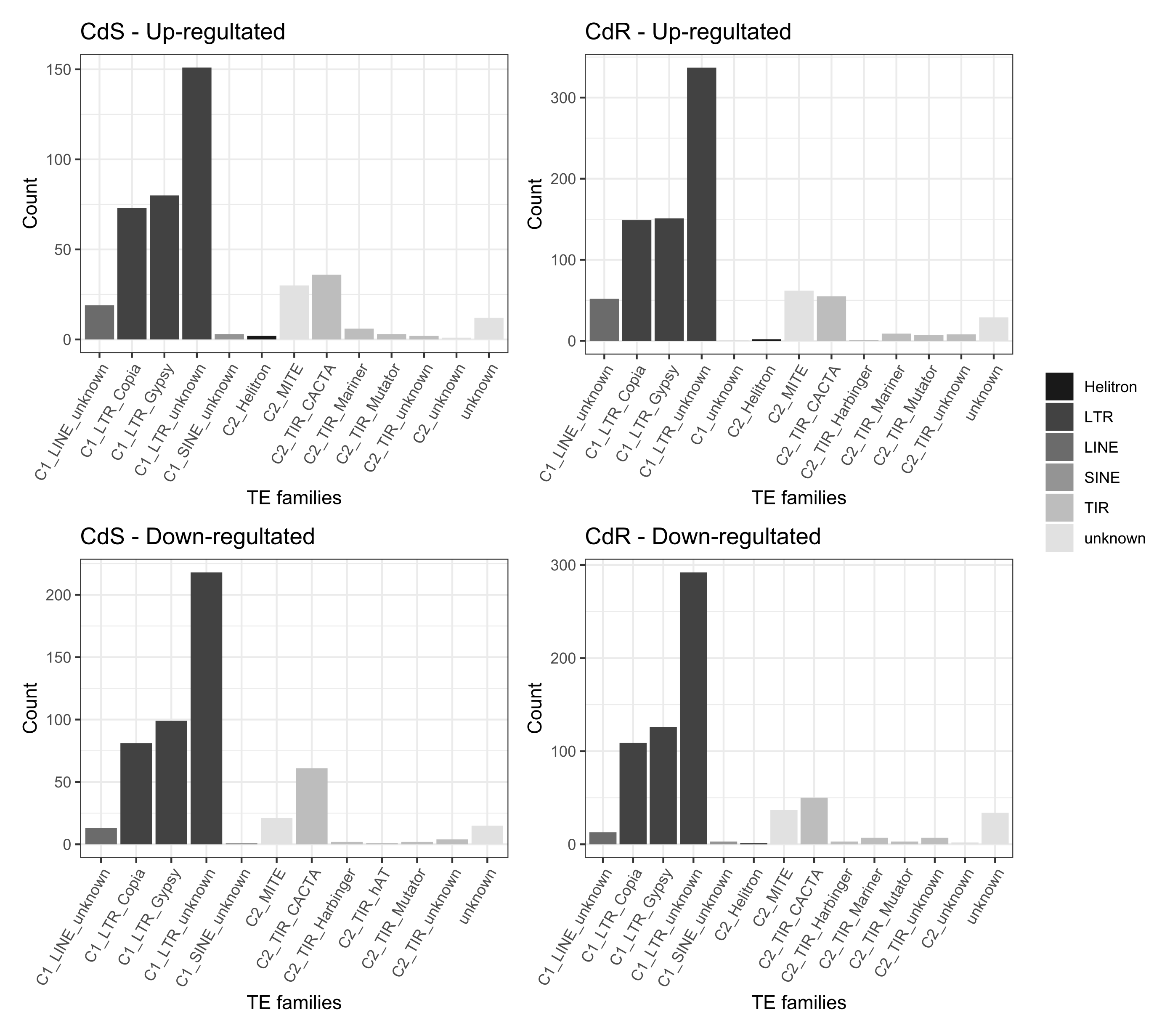


#### Supplementary Figure 3. Distribution of differentially expressed transposable elements. Element names are in the form X_Y_Z where X corresponds to the element class with C1 for retrotransposon and C2 for DNA-transposon. Y corresponds to the order of the element and Z its superfamily.


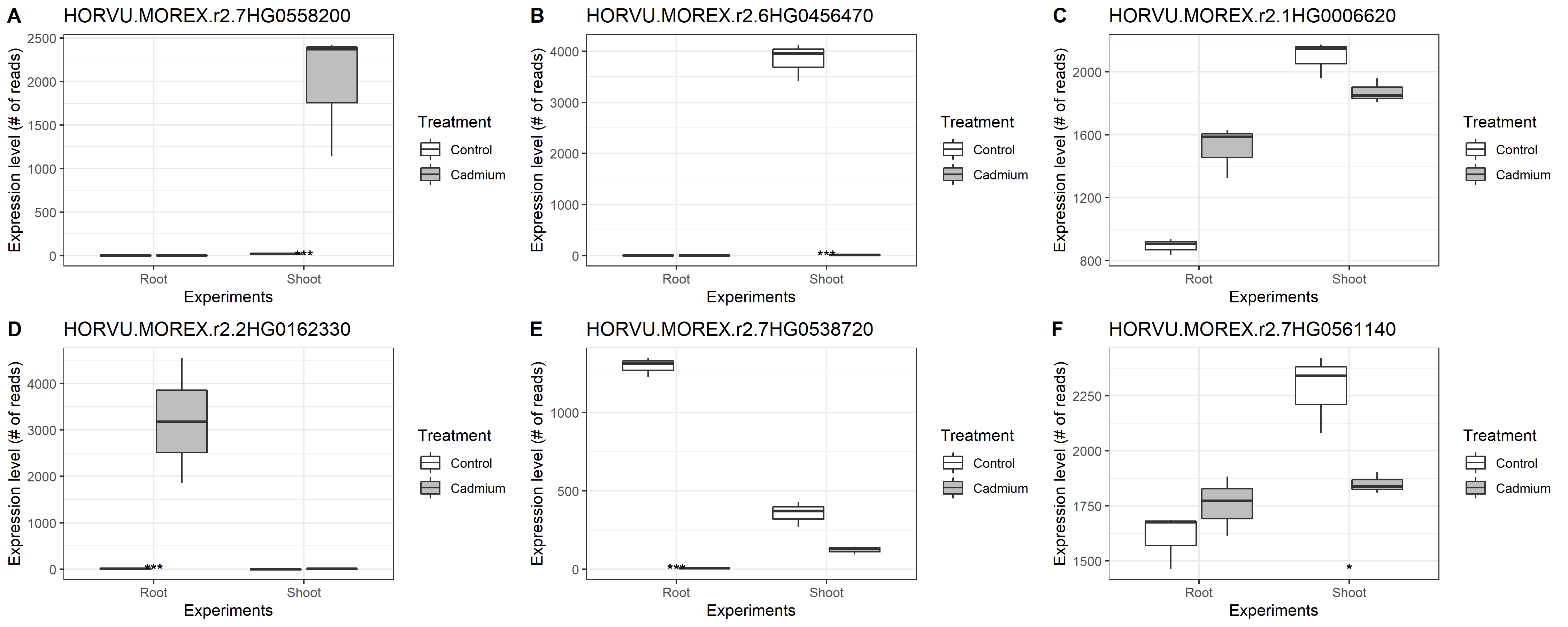


#### Supplementary Figure 4. Expression of selected candidates for qPCR analysis. Box plot showing the expression level of a gene as count of reads for both root and shoot tissues in control and cadmium treated conditions. The difference between control and treatment was statistically tested and the adjusted P-value < 0.05 is displayed as an asterisk (*), similarly P-value < 0.01 and P-value < 0.005 are displayed as (**) and (***) respectively.


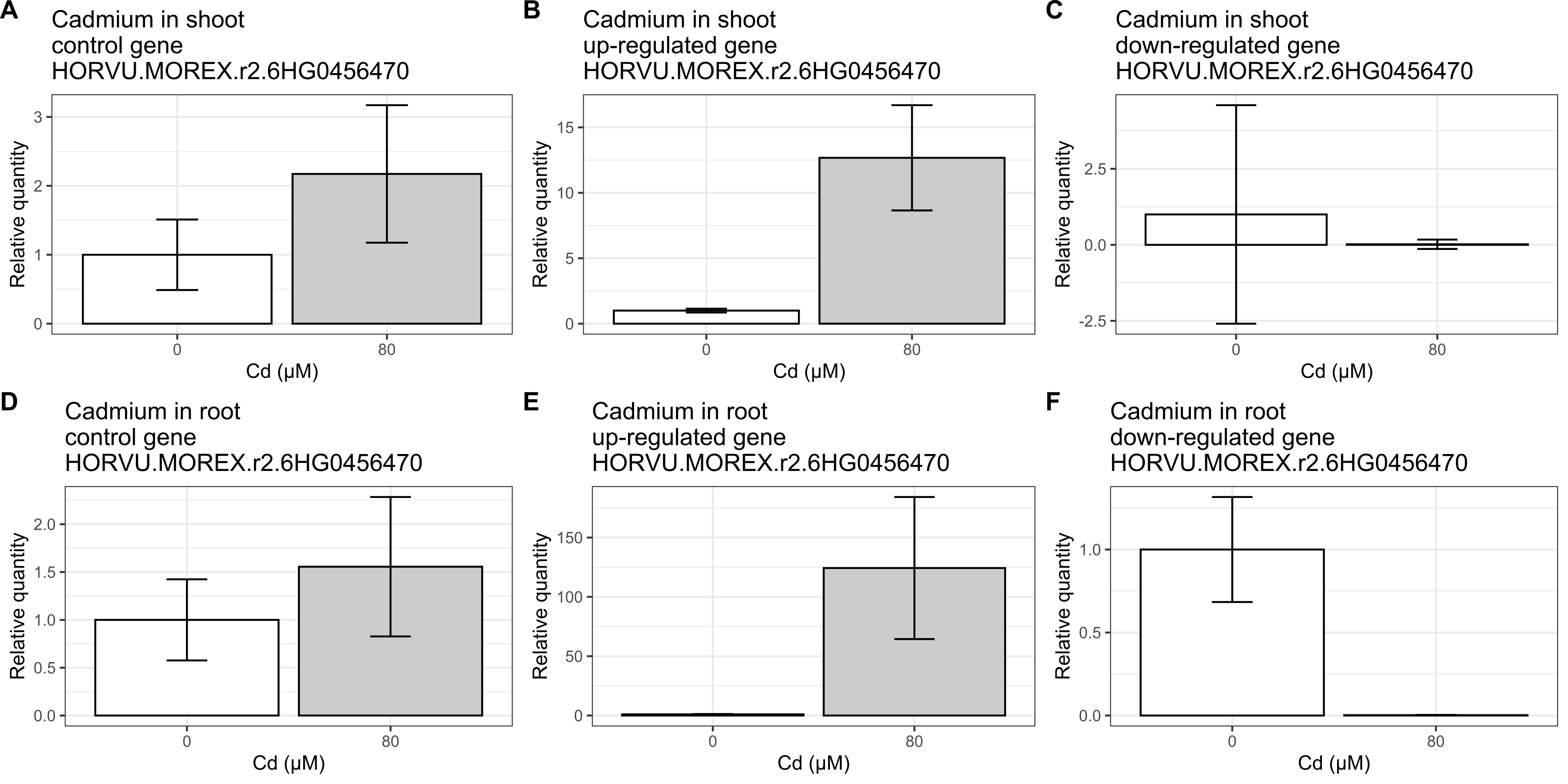


#### Supplementary Figure 5. RT-qPCR validation of RNA-seq for selected candidates. Relative expression of selected candidate genes. Each bar in the graph is the average of three technical repetitions for each of three biological replicates.
