# Supplementary material for: Transcriptome Response to Cadmium Exposure in Barley (Hordeum vulgare L.)
Source: Front Plant Sci. 2021 Jul 15;12:629089. doi: 10.3389/fpls.2021.629089 (PMC8321094; doi:10.3389/fpls.2021.629089)
Supplement: Supplementary file 2 [file Data_Sheet_2.DOCX]

Supplementary Material – Figures 6-22


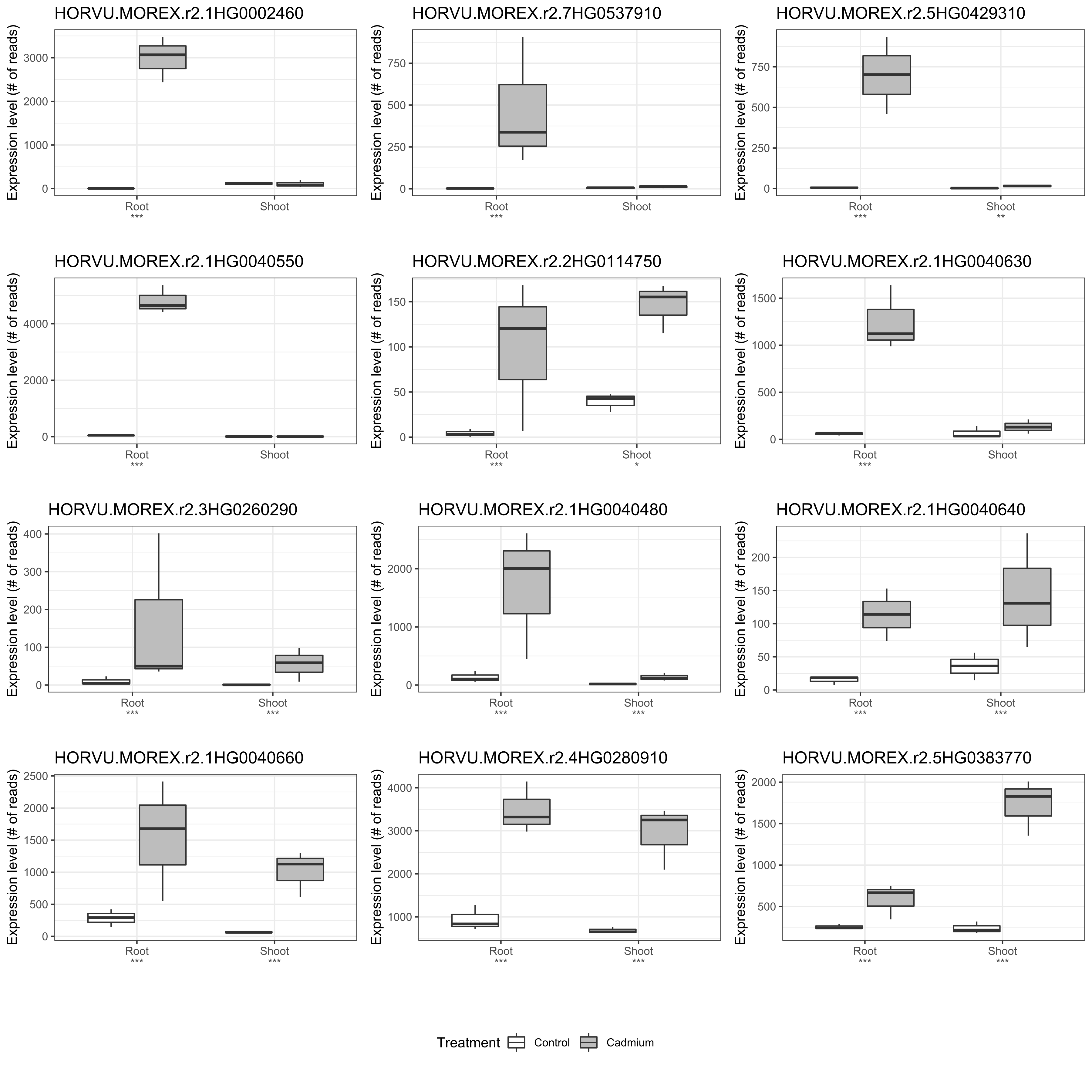


**Supplementary Figure 6.** Cd-dependent upregulation of transcripts encoding glutathione-
S-transferase (GST). Box plot showing the expression level of a gene as count of reads for both root and shoot tissues in control and cadmium treated conditions. The difference between control and treatment was statistically tested and the adjusted P-value < 0.05 is displayed as an asterisk (*), similarly P-value < 0.01 and P-value < 0.005 are displayed as (**) and (***) respectively.


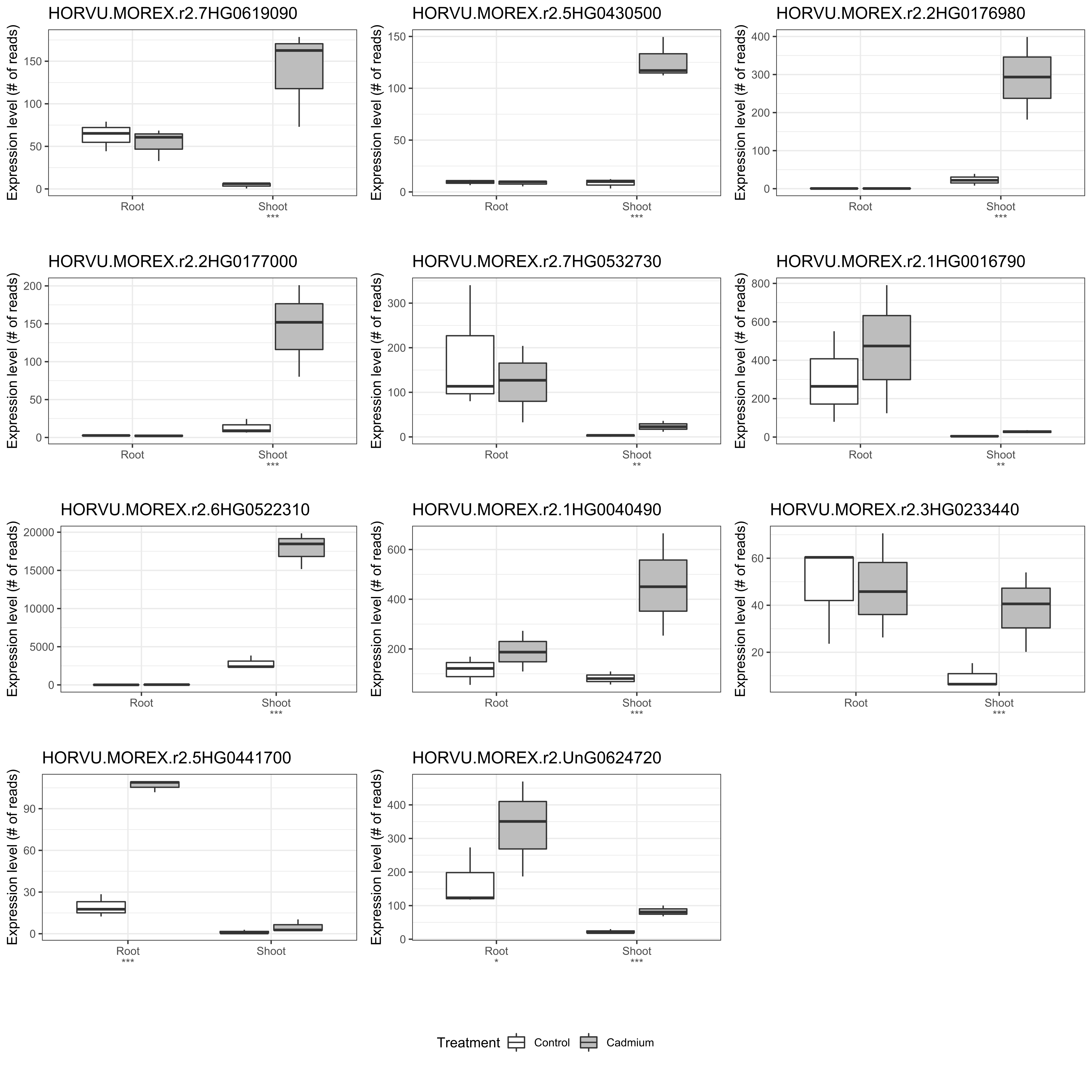
 **Supplementary Figure 7.** Cd-dependent upregulation of transcripts encoding glutathione-
S-transferase (GST). Box plot showing the expression level of a gene as count of reads for both root and shoot tissues in control and cadmium treated conditions. The difference between control and treatment was statistically tested and the adjusted P-value < 0.05 is displayed as an asterisk (*), similarly P-value < 0.01 and P-value < 0.005 are displayed as (**) and (***) respectively.

**
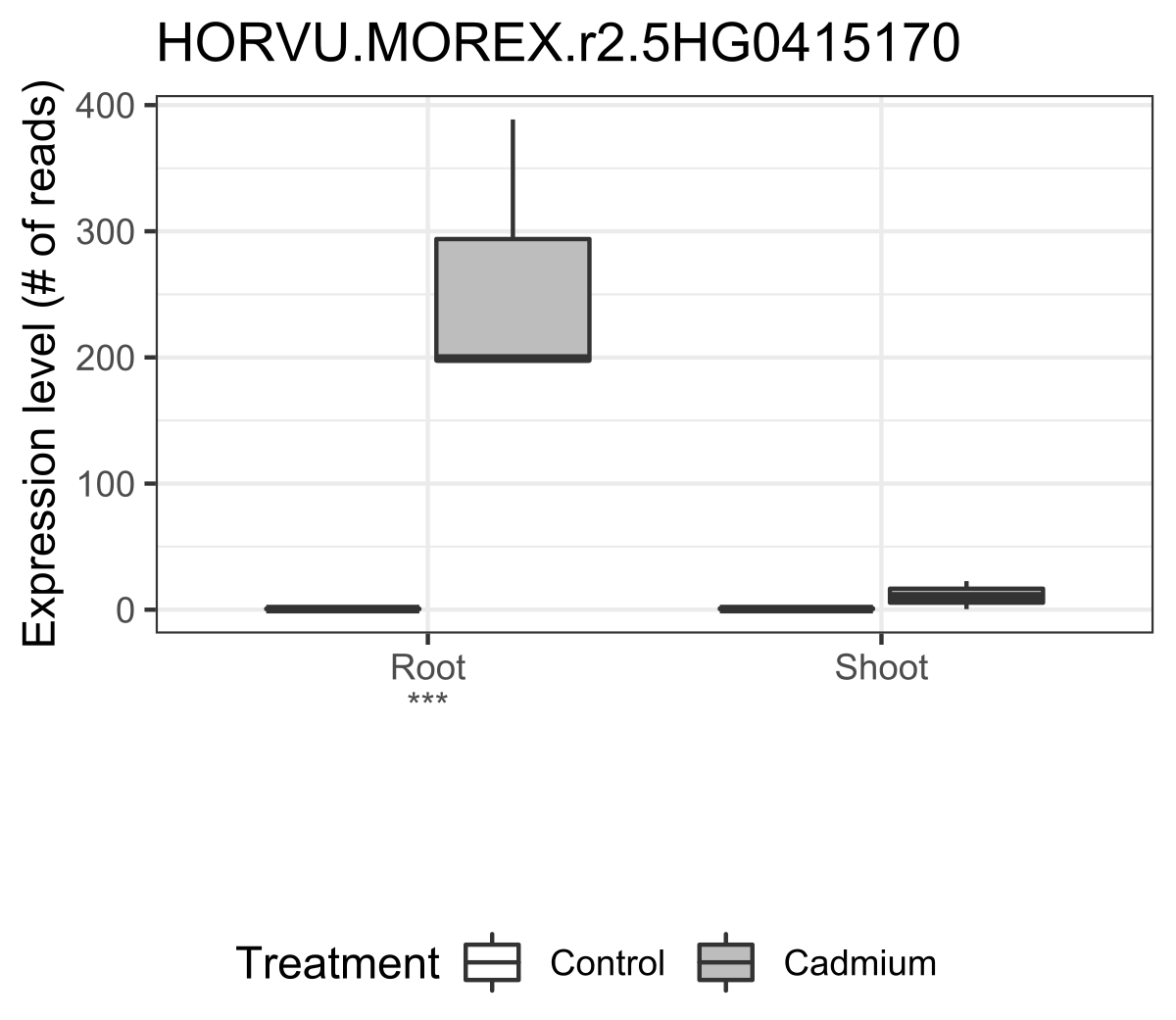
Supplementary Figure 8.** Cd-dependent upregulation of transcript encoding purple acid phosphatase (PAP). Box plot showing the expression level of a gene as count of reads for both root and shoot tissues in control and cadmium treated conditions. The difference between control and treatment was statistically tested and the adjusted P-value < 0.05 is displayed as an asterisk (*), similarly P-value < 0.01 and P-value < 0.005 are displayed as (**) and (***) respectively.


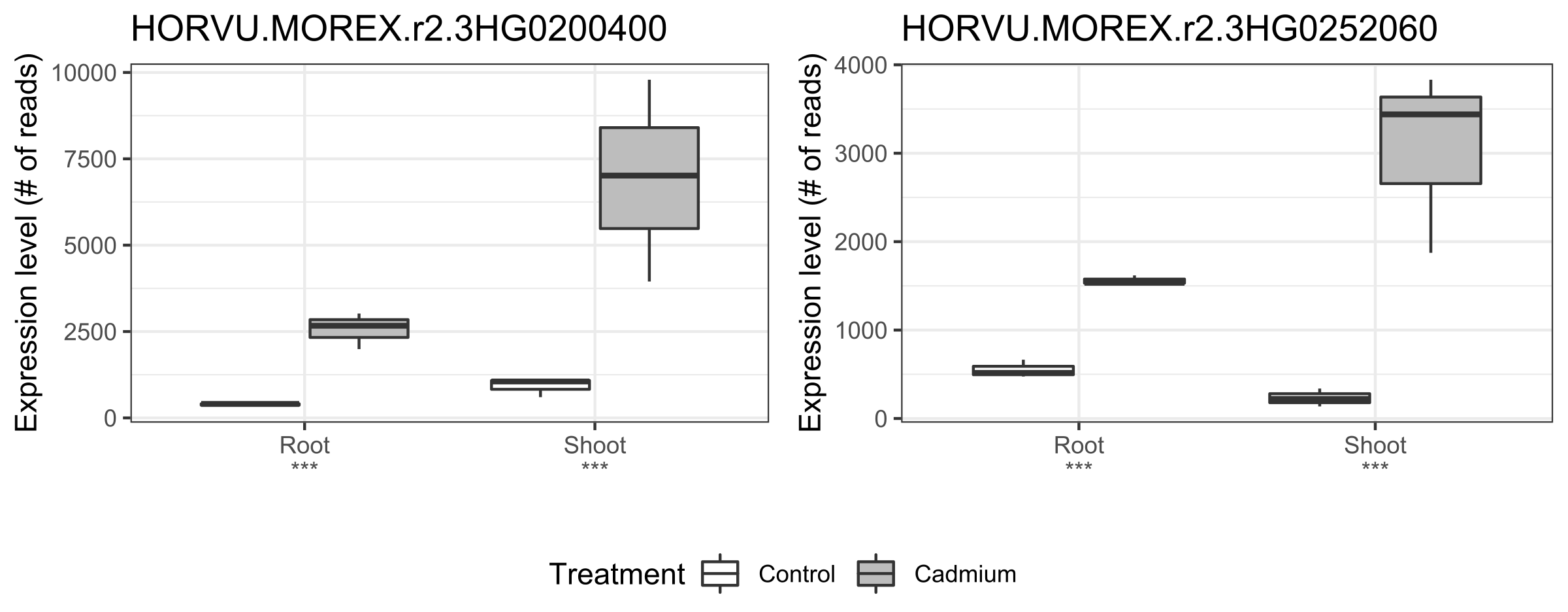


**Supplementary Figure 9.** Cd-dependent upregulation of transcripts encoding multidrug resistance-associated protein 3 (MRP3). Box plot showing the expression level of a gene as count of reads for both root and shoot tissues in control and cadmium treated conditions. The difference between control and treatment was statistically tested and the adjusted P-value < 0.05 is displayed as an asterisk (*), similarly P-value < 0.01 and P-value < 0.005 are displayed as (**) and (***) respectively.


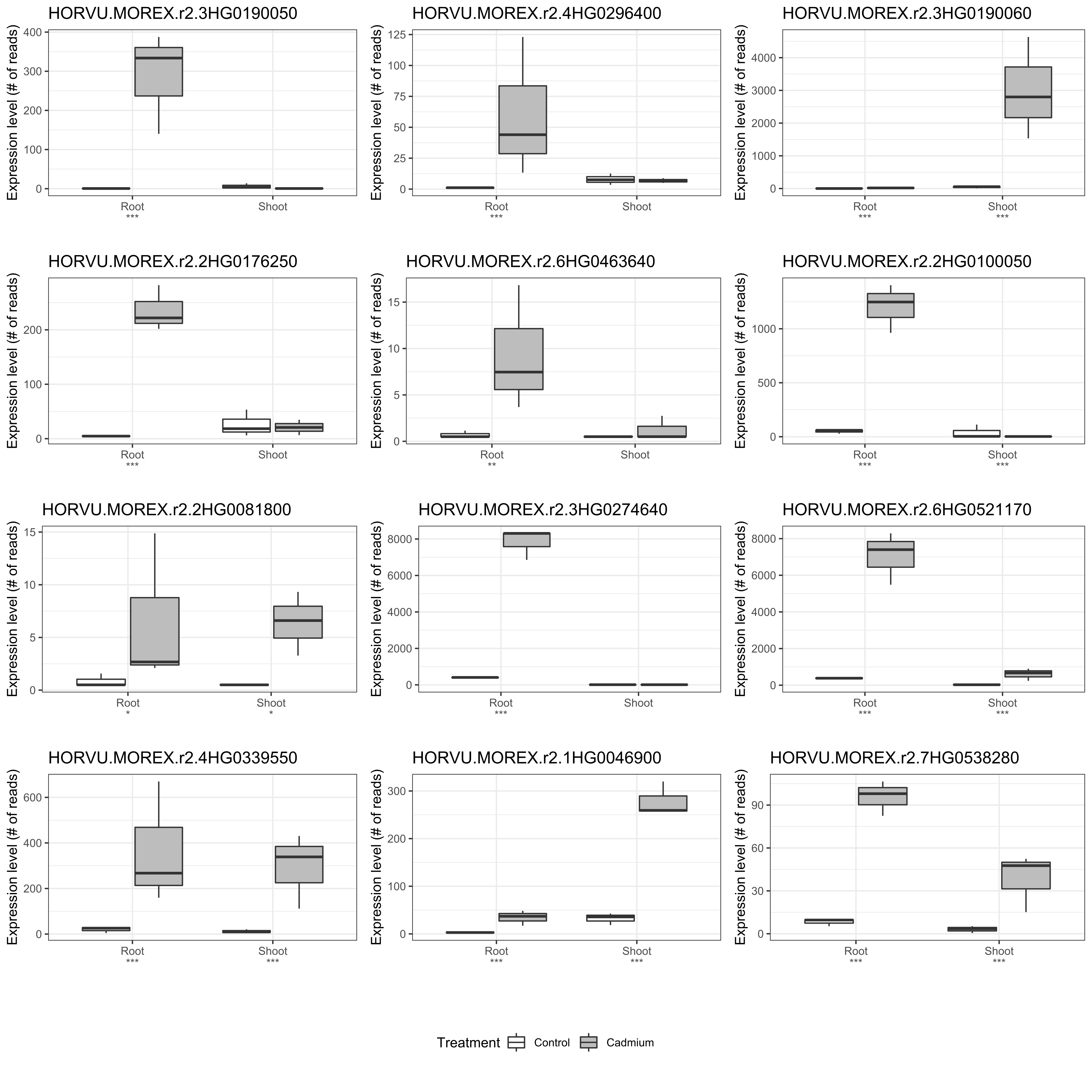


**Supplementary Figure 10.** Cd-dependent upregulation of transcripts encoding cytochrome P450. Box plot showing the expression level of a gene as count of reads for both root and shoot tissues in control and cadmium treated conditions. The difference between control and treatment was statistically tested and the adjusted P-value < 0.05 is displayed as an asterisk (*), similarly P-value < 0.01 and P-value < 0.005 are displayed as (**) and (***) respectively.


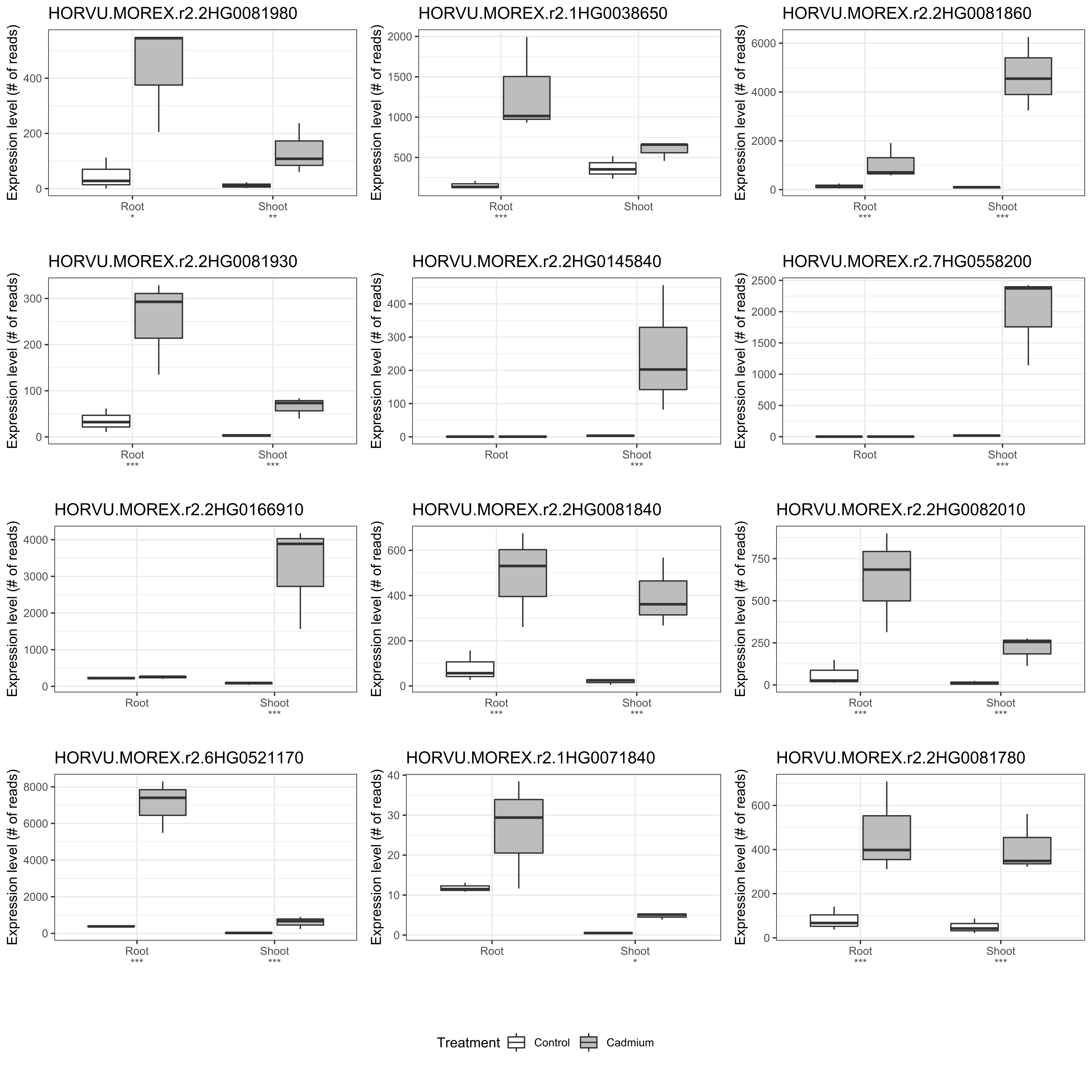


**Supplementary Figure 11.** Cd-dependent upregulation of transcripts encoding cytochrome P450. Box plot showing the expression level of a gene as count of reads for both root and shoot tissues in control and cadmium treated conditions. The difference between control and treatment was statistically tested and the adjusted P-value < 0.05 is displayed as an asterisk (*), similarly P-value < 0.01 and P-value < 0.005 are displayed as (**) and (***) respectively.


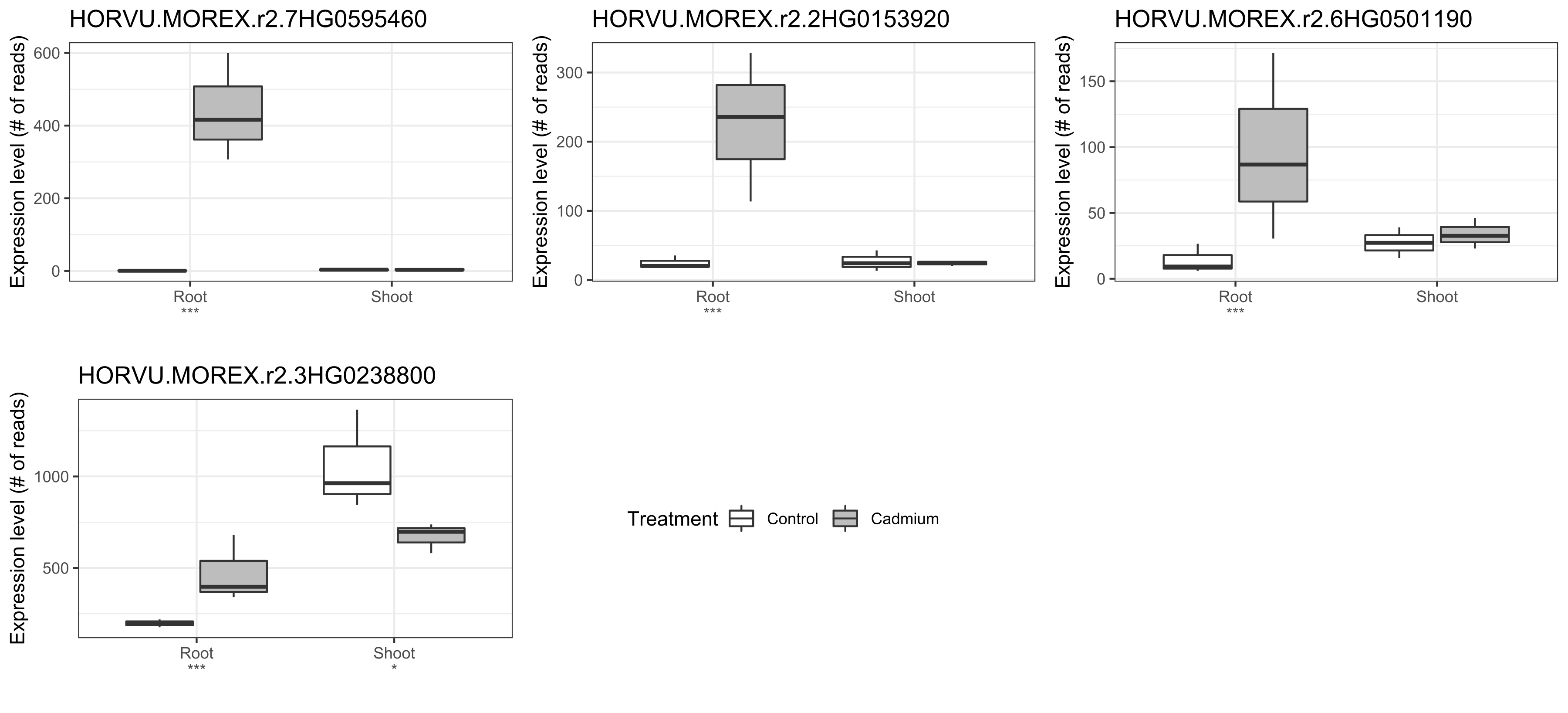
**Supplementary Figure 13.** Cd-dependent upregulation of transcripts encoding OXIDATIVE STRESS 3 homologs. Box plot showing the expression level of a gene as count of reads for both root and shoot tissues in control and cadmium treated conditions. The difference between control and treatment was statistically tested and the adjusted P-value < 0.05 is displayed as an asterisk (*), similarly P-value < 0.01 and P-value < 0.005 are displayed as (**) and (***) respectively.

**
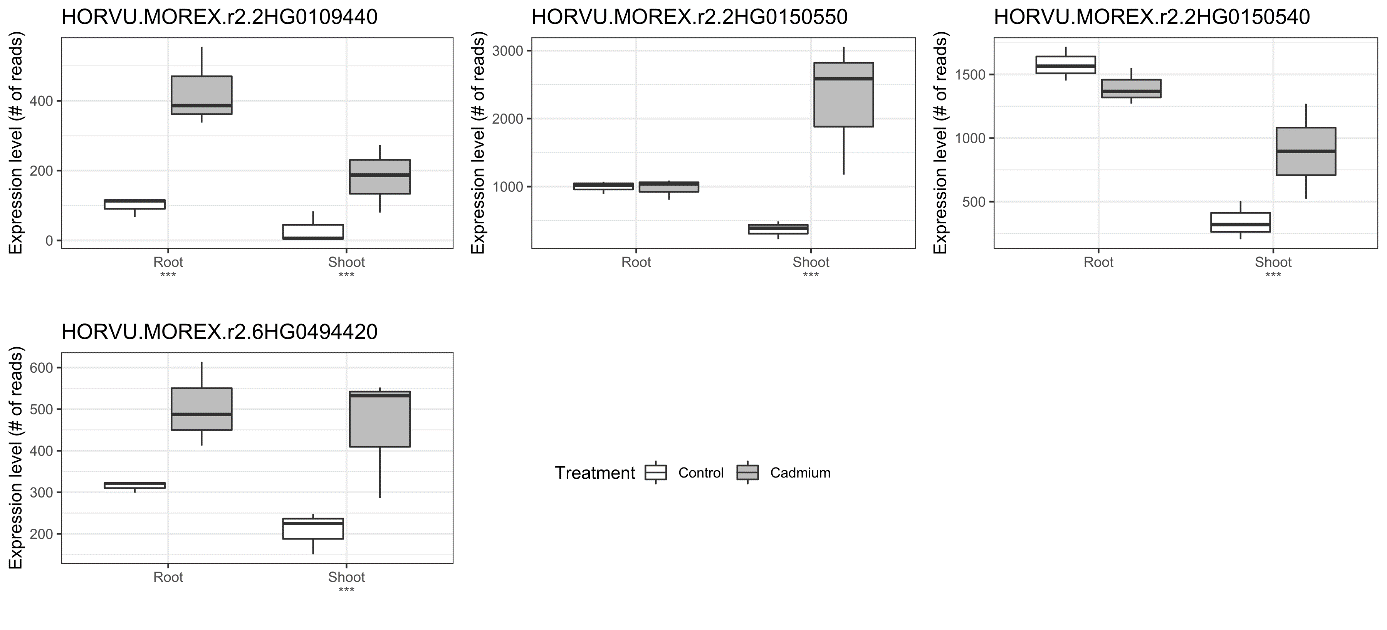
**

**Supplementary Figure 13.** Cd-dependent upregulation of transcripts encoding phenylalanine ammonia lyase (PAL). Box plot showing the expression level of a gene as count of reads for both root and shoot tissues in control and cadmium treated conditions. The difference between control and treatment was statistically tested and the adjusted P-value < 0.05 is displayed as an asterisk (*), similarly P-value < 0.01 and P-value < 0.005 are displayed as (**) and (***) respectively.

**
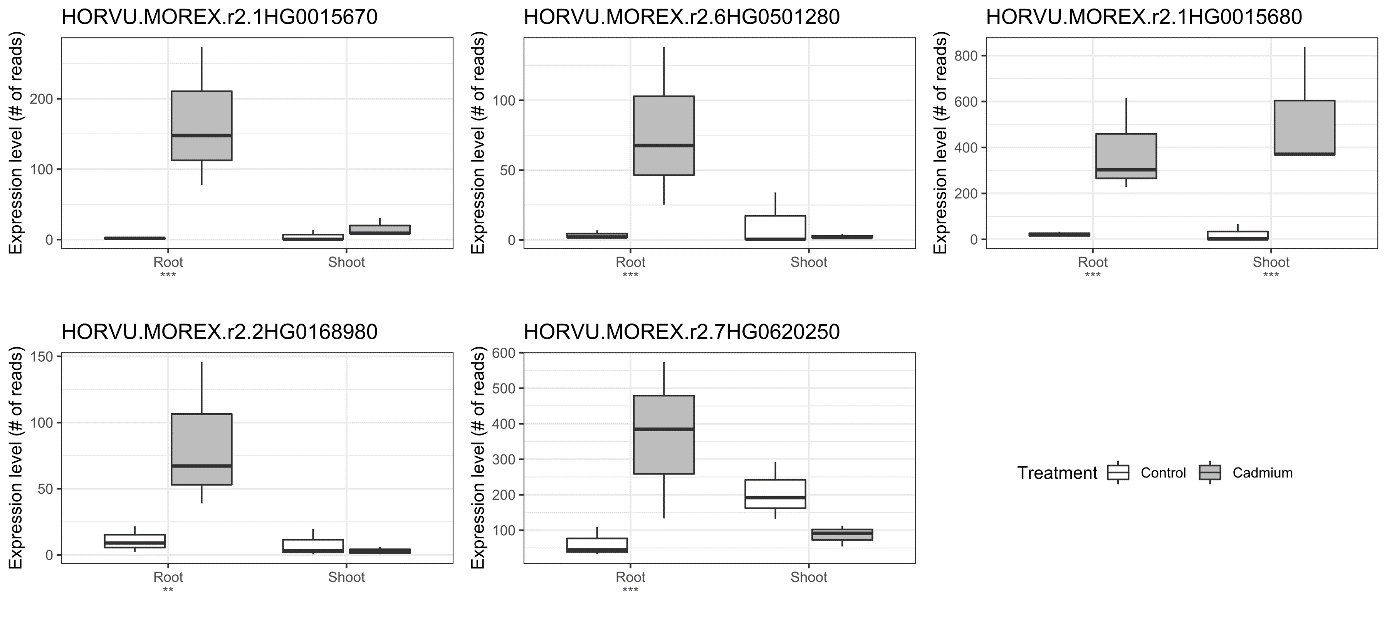
**

**Supplementary Figure 14.** Cd-dependent upregulation of transcripts encoding shikimate O-hydroxycinnamoyltransferase (HCT). Box plot showing the expression level of a gene as count of reads for both root and shoot tissues in control and cadmium treated conditions. The difference between control and treatment was statistically tested and the adjusted P-value < 0.05 is displayed as an asterisk (*), similarly P-value < 0.01 and P-value < 0.005 are displayed as (**) and (***) respectively.


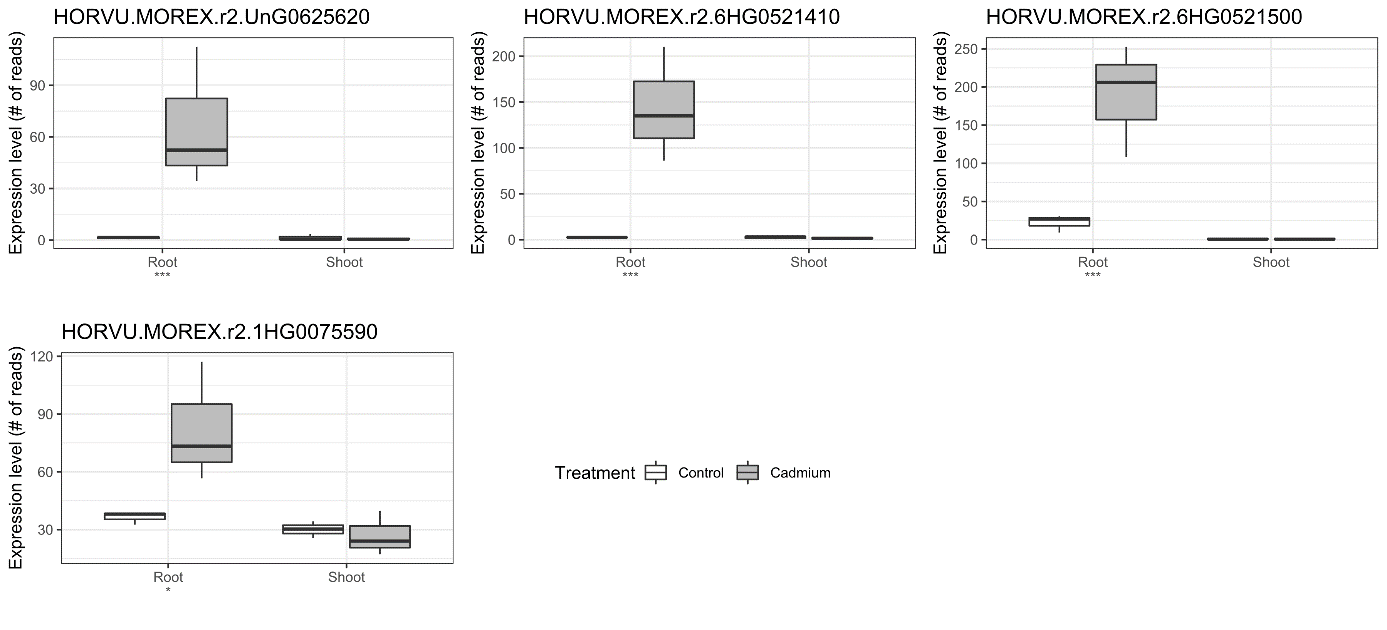


**Supplementary Figure 15.** Cd-dependent upregulation of transcripts encoding cinnamoyl-CoA reductase (CCR). Box plot showing the expression level of a gene as count of reads for both root and shoot tissues in control and cadmium treated conditions. The difference between control and treatment was statistically tested and the adjusted P-value < 0.05 is displayed as an asterisk (*), similarly P-value < 0.01 and P-value < 0.005 are displayed as (**) and (***) respectively.

**
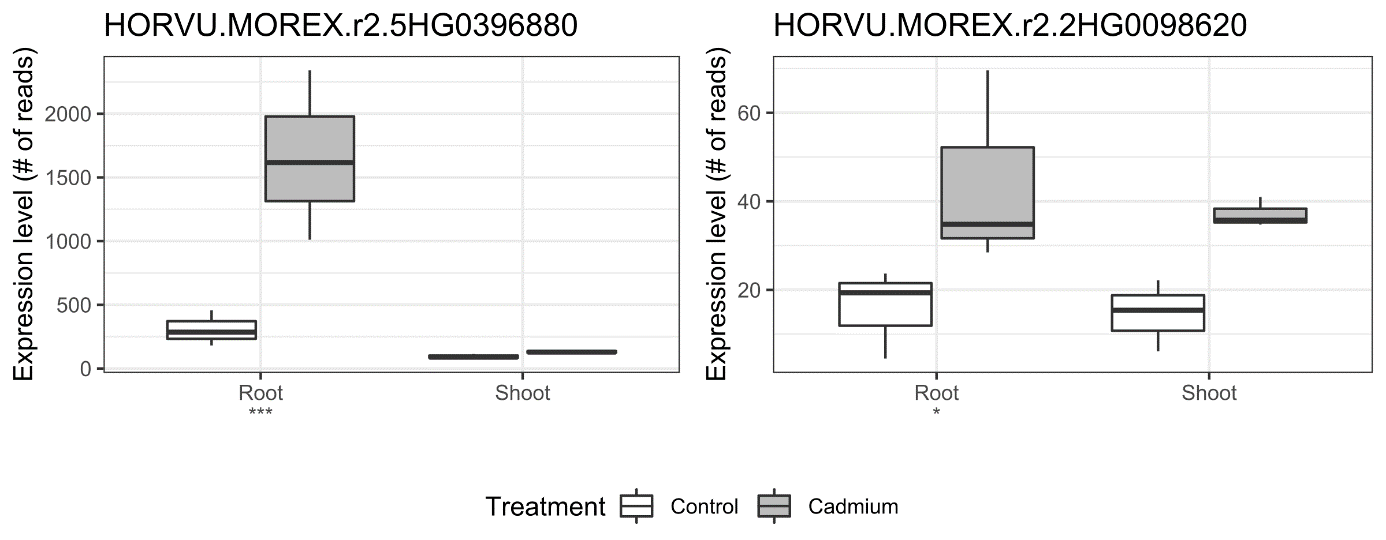
**

**Supplementary Figure 16.** Cd-dependent upregulation of transcripts encoding cinnamyl alcohol dehydrogenase (CAD). Box plot showing the expression level of a gene as count of reads for both root and shoot tissues in control and cadmium treated conditions. The difference between control and treatment was statistically tested and the adjusted P-value < 0.05 is displayed as an asterisk (*), similarly P-value < 0.01 and P-value < 0.005 are displayed as (**) and (***) respectively.


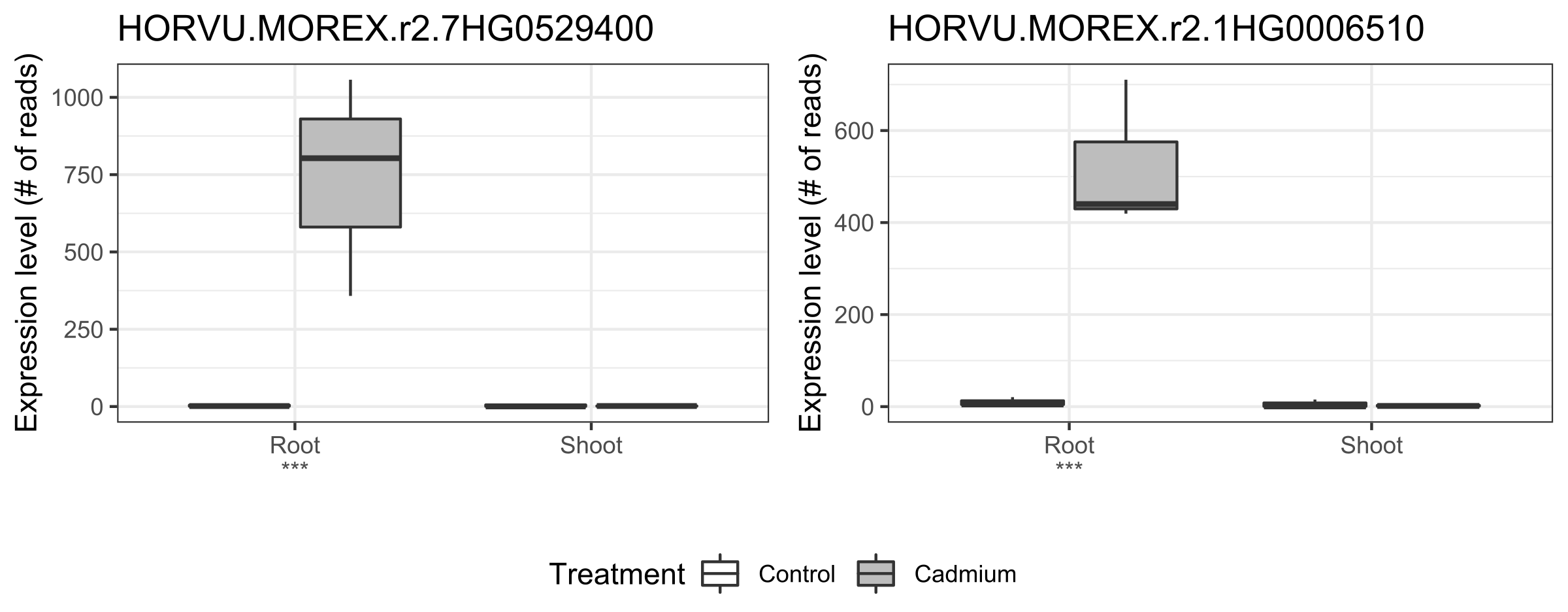


**Supplementary Figure 17.** Cd-dependent upregulation of transcripts encoding laccase 7 (LAC7). Box plot showing the expression level of a gene as count of reads for both root and shoot tissues in control and cadmium treated conditions. The difference between control and treatment was statistically tested and the adjusted P-value < 0.05 is displayed as an asterisk (*), similarly P-value < 0.01 and P-value < 0.005 are displayed as (**) and (***) respectively.


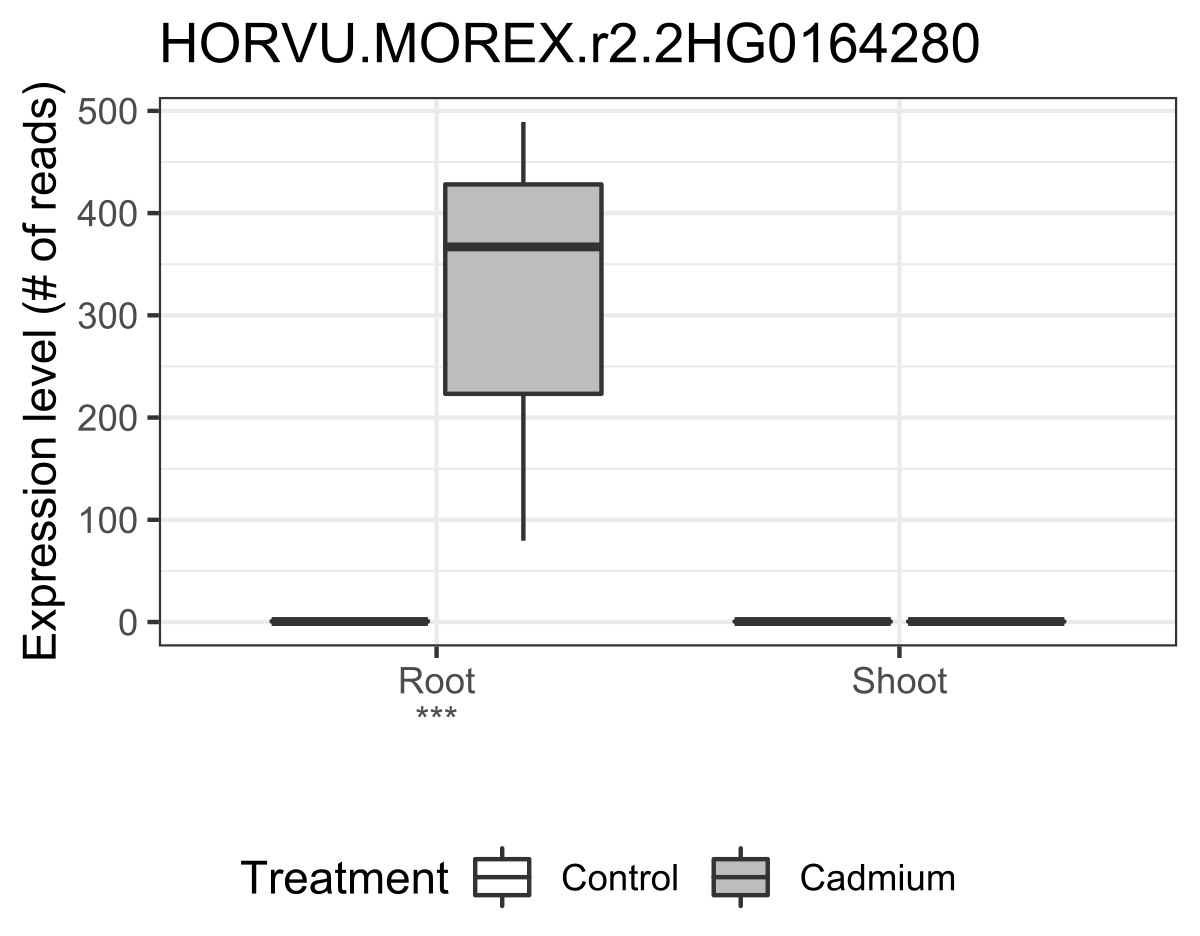

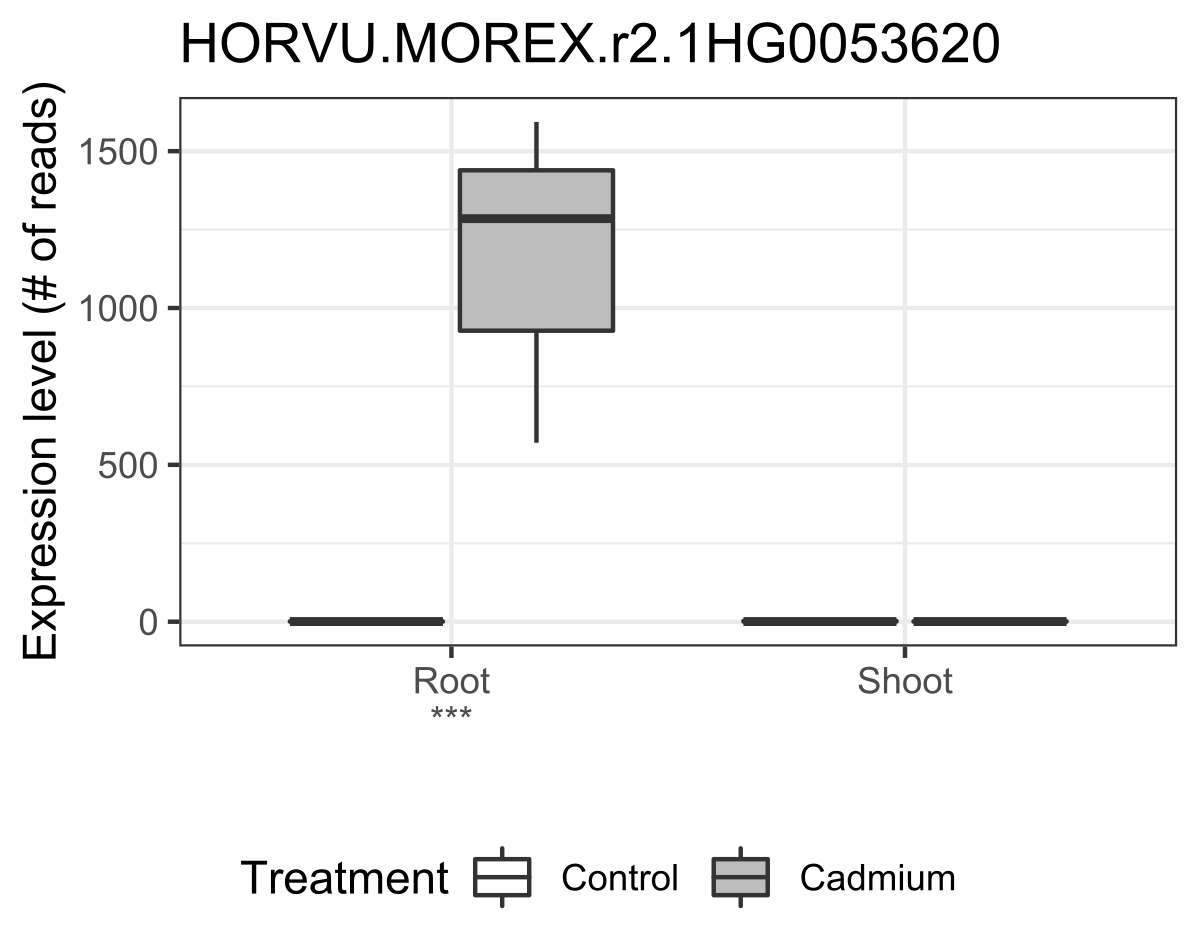
 **Supplementary Figure 18.** Cd-dependent upregulation of transcripts encoding homologs of wound induced protein 1 (HORVU.MOREX.r2.1HG0053620) or leucine-rich repeat receptor-like protein kinase family protein (HORVU.MOREX.r2.2HG0164280). Box plot showing the expression level of a gene as count of reads for both root and shoot tissues in control and cadmium treated conditions. The difference between control and treatment was statistically tested and the adjusted P-value < 0.05 is displayed as an asterisk (*), similarly P-value < 0.01 and P-value < 0.005 are displayed as (**) and (***) respectively.

**Supplementary Figure 19.** Cd-dependent upregulation of transcripts encoding peroxidase (POD). Box plot showing the expression level of a gene as count of reads for both root and shoot tissues in control and cadmium treated conditions. The difference between control and treatment was statistically tested and the adjusted P-value < 0.05 is displayed as an asterisk (*), similarly P-value < 0.01 and P-value < 0.005 are displayed as (**) and (***) respectively.


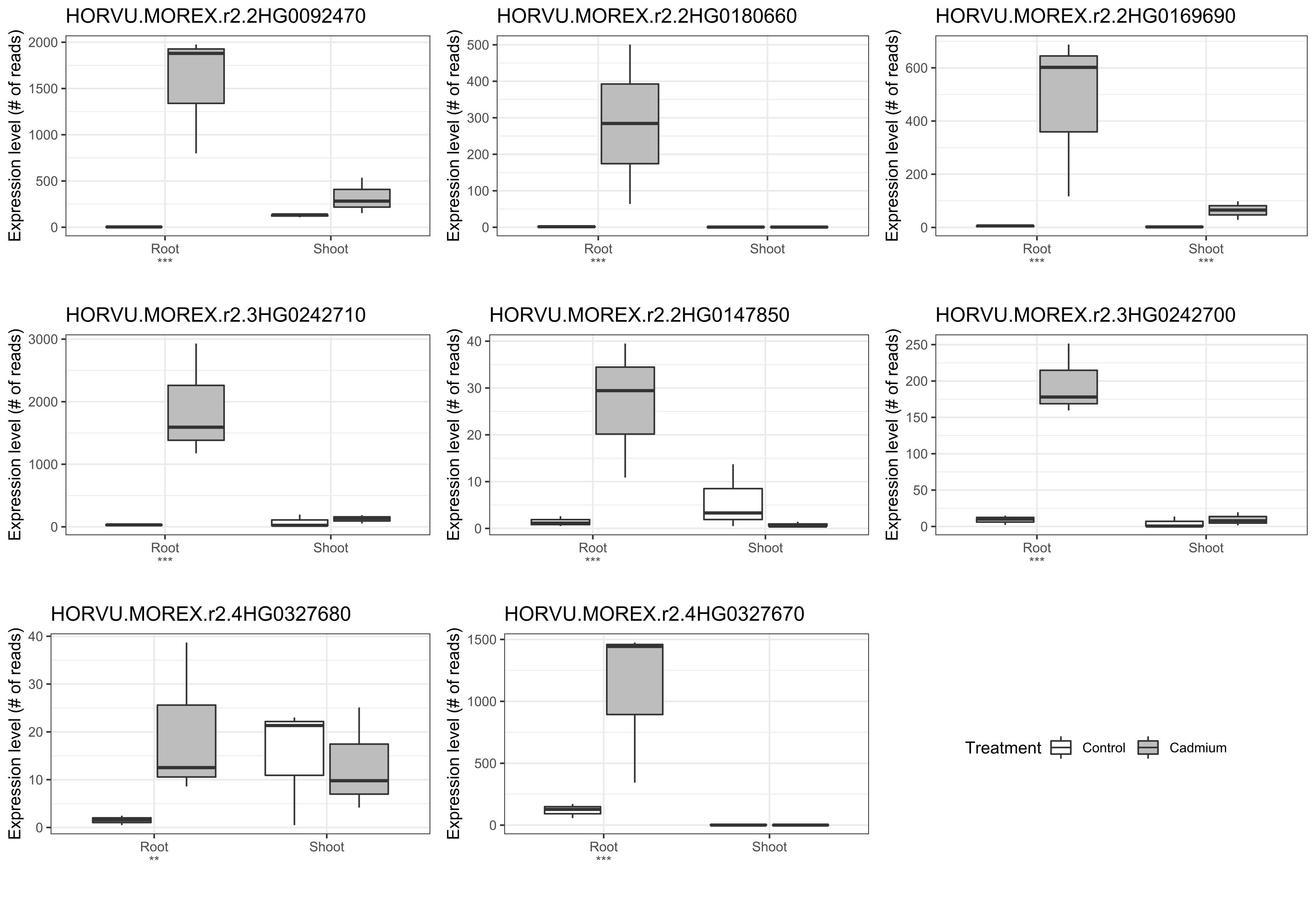


**
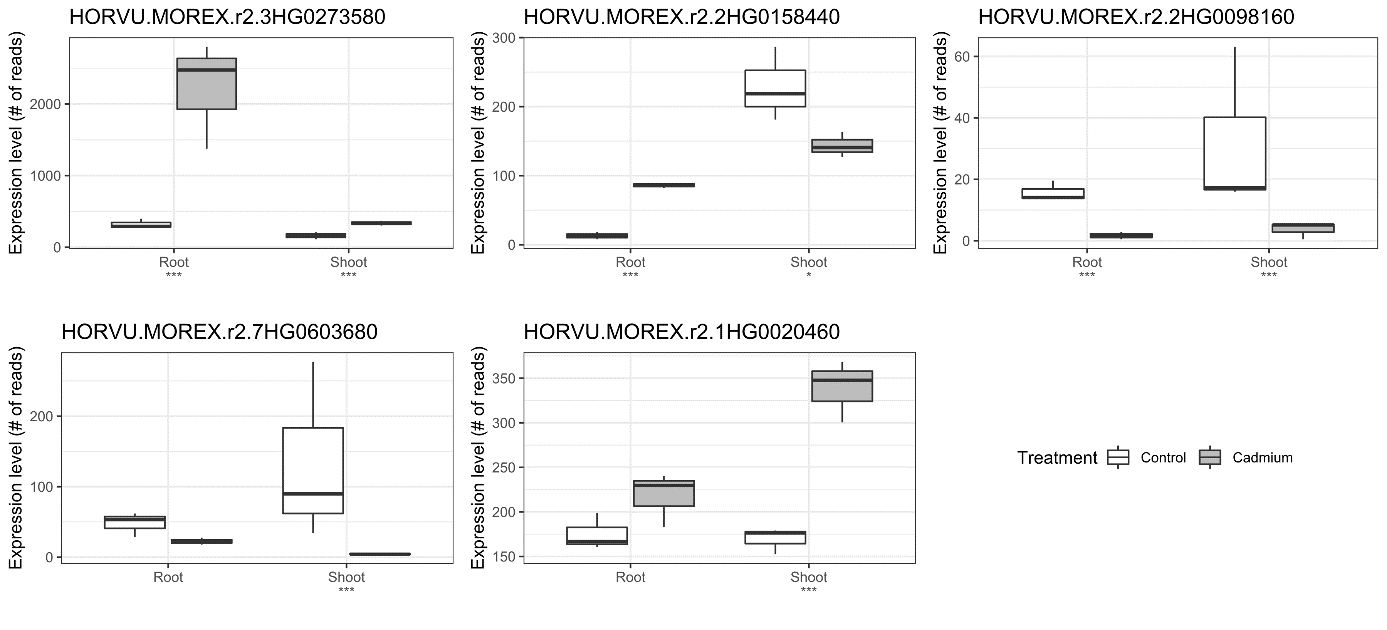
**

**Supplementary Figure 20.** Cd-dependent upregulation of transcripts encoding ZRT/IRT‐like protein (ZIP) family transporters - *HvZIP1* (*HORVU.MOREX.r2.3HG0273580*), *HvZIP3* (*HORVU.MOREX.r2.2HG0158440*) and *HvZIP6* (*HORVU.MOREX.r2.1HG0020460*). Box plot showing the expression level of a gene as count of reads for both root and shoot tissues in control and cadmium treated conditions. The difference between control and treatment was statistically tested and the adjusted P-value < 0.05 is displayed as an asterisk (*), similarly P-value < 0.01 and P-value < 0.005 are displayed as (**) and (***) respectively.


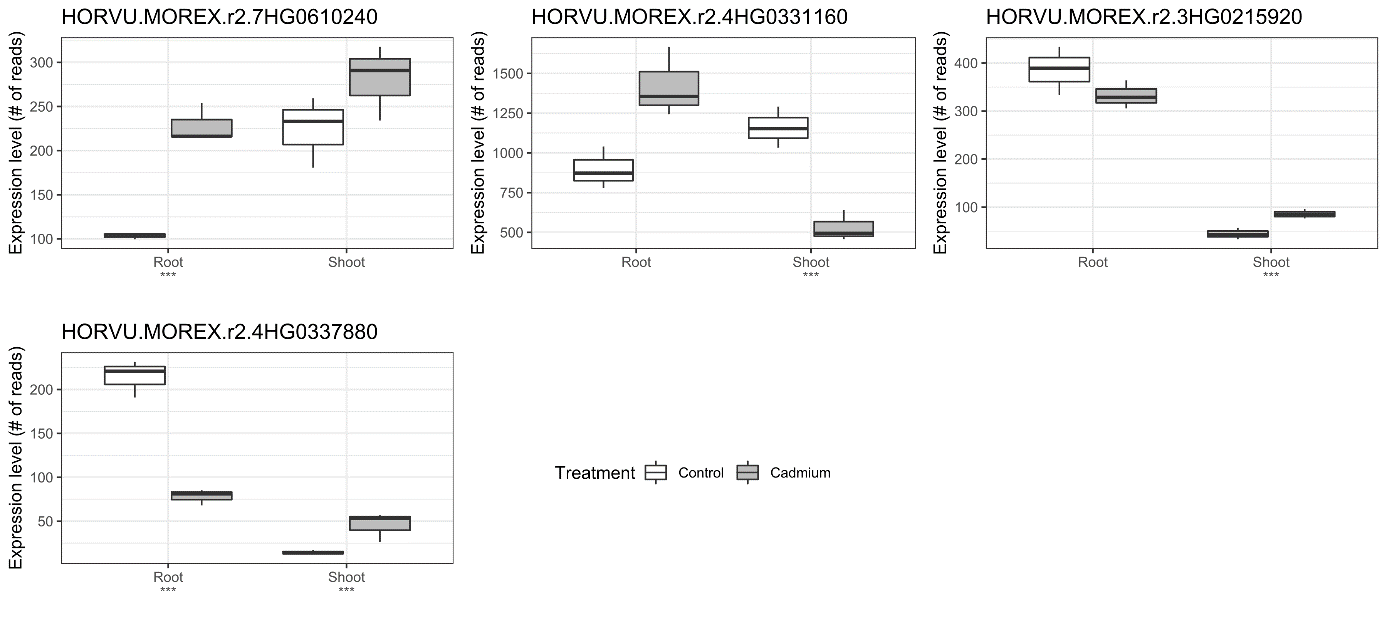


**Supplementary Figure 21.** Cd-dependent upregulation of transcripts encoding NATURAL RESISTANCE ASSOCIATED MACROPHAGE PROTEIN (NRAMP) - *HvNRAMP5* (*HORVU.MOREX.r2.4HG0337880*), *HvNRAMP2* (*HORVU.MOREX.r2.4HG0331160*), *HvNRAMP1* (*HORVU.MOREX.r2.7HG0610240*) and *HvNRAMP6* (*HORVU.MOREX.r2.3HG0215920*). Box plot showing the expression level of a gene as count of reads for both root and shoot tissues in control and cadmium treated conditions. The difference between control and treatment was statistically tested and the adjusted P-value < 0.05 is displayed as an asterisk (*), similarly P-value < 0.01 and P-value < 0.005 are displayed as (**) and (***) respectively.


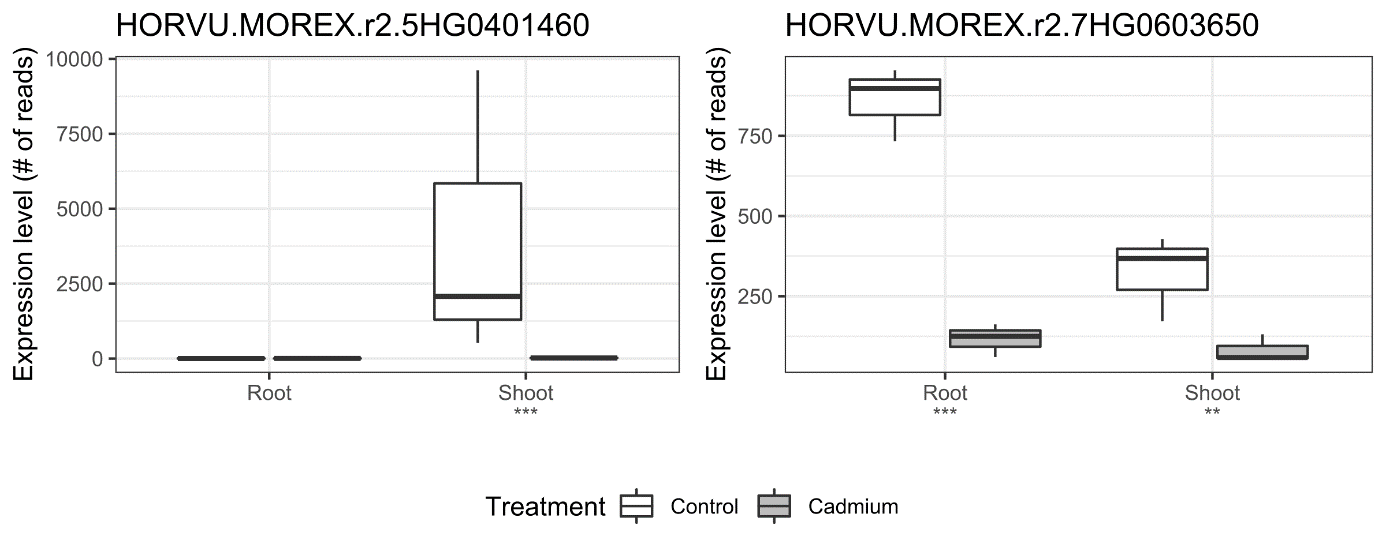


**Supplementary Figure 22.** Cd-dependent upregulation of transcripts encoding HEAVY METAL ATPASE (HMA)- *HvHMA1* (HORVU.MOREX.r2.5HG0401460) and *HvHMA3* (*HORVU.MOREX.r2.7HG0603650*). Box plot showing the expression level of a gene as count of reads for both root and shoot tissues in control and cadmium treated conditions. The difference between control and treatment was statistically tested and the adjusted P-value < 0.05 is displayed as an asterisk (*), similarly P-value < 0.01 and P-value < 0.005 are displayed as (**) and (***) respectively.
