# Supplementary material for: Transcriptome Response to Cadmium Exposure in Barley (Hordeum vulgare L.)
Source: Front Plant Sci. 2021 Jul 15;12:629089. doi: 10.3389/fpls.2021.629089 (PMC8321094; doi:10.3389/fpls.2021.629089)
Supplement: Supplementary file 3 [file Data_Sheet_3.docx]

Supplementary Material – Figure 23


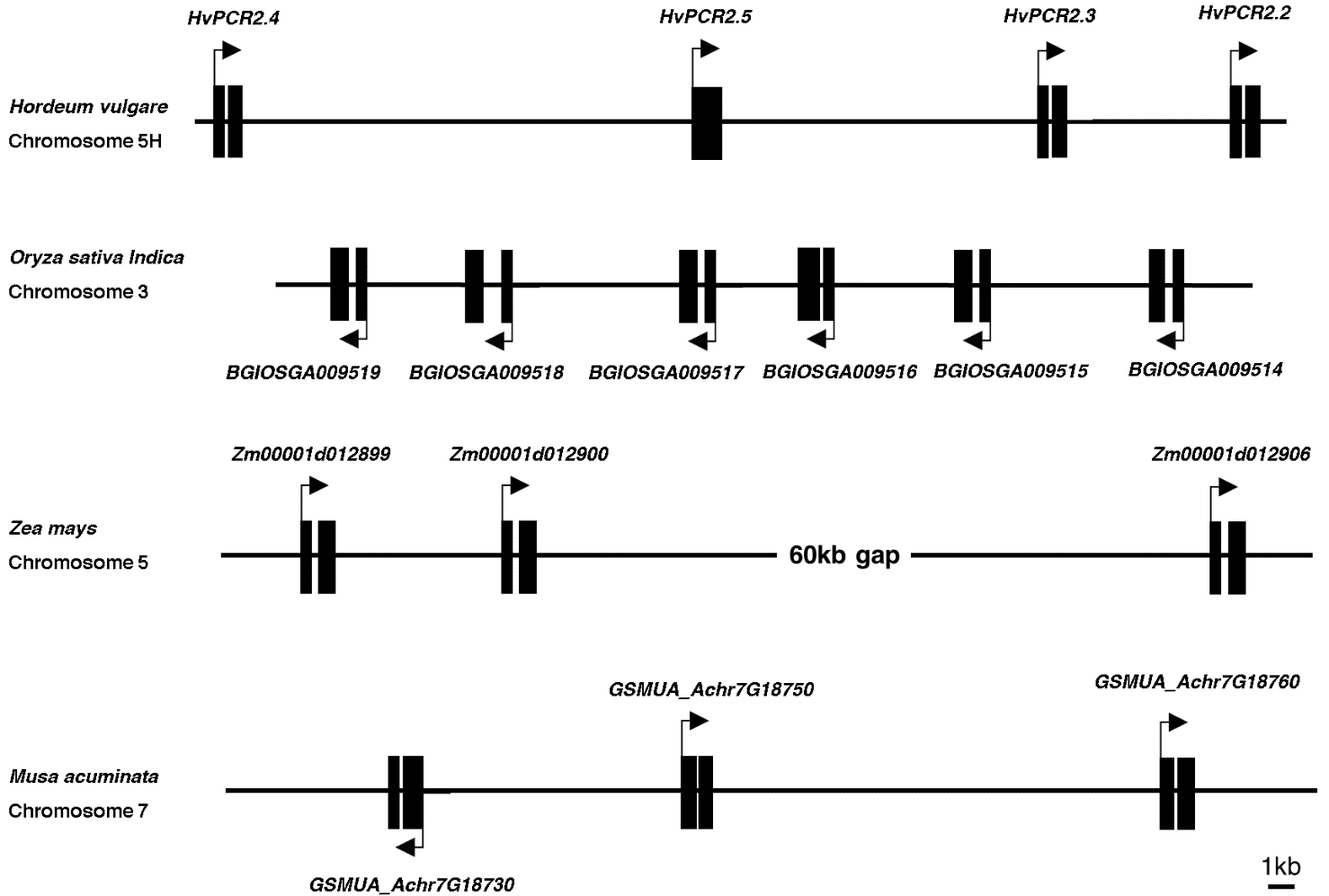


**Supplementary Figure 23.** Schematic diagram showing the arrangement of *PCR2* homologs in genomic regions of barley (*Hordeum vulgare*; *HvPCR2.2-4*), rice (*Oryza sativa,* *BGIOSGA009514-19)*, corn (*Zea mays; Zm00001d012899, Zm00001d012900* and *Zm00001d012906*) and banana (*Musa acuminata; GSMUA_Achr7G18730, GSMUA_Achr7G18750* and *GSMUA_Achr7G18760*).
