# Supplementary material for: Transcriptome Response to Cadmium Exposure in Barley (Hordeum vulgare L.)
Source: Front Plant Sci. 2021 Jul 15;12:629089. doi: 10.3389/fpls.2021.629089 (PMC8321094; doi:10.3389/fpls.2021.629089)
Supplement: Supplementary file 4 [file Data_Sheet_4.docx]

Supplementary Material – Tables 1-5

#### Supplementary Table 1. Comparison of initial and final root length on control and treated plants.

| Plant | Treatment | Initial root length | Final root length | Increment |
| --- | --- | --- | --- | --- |
| 1 | control | 1.7 | 7.4 | 5.7 |
| 2 | control | 1.8 | 6.9 | 5.1 |
| 3 | control | 1.7 | 5.3 | 3.6 |
| 4 | control | 1.2 | 6.5 | 5.3 |
| 5 | control | 1.3 | 5.5 | 4.2 |
| 6 | control | 1.1 | 6.7 | 5.6 |
| 7 | treated | 1.6 | 4.3 | 2.7 |
| 8 | treated | 1.2 | 4.1 | 2.9 |
| 9 | treated | 1.4 | 4 | 2.6 |
| 10 | treated | 1.4 | 4.1 | 2.7 |
| 11 | treated | 2 | 4 | 2 |
| 12 | treated | 2.1 | 4.2 | 2.1 |

#### Supplementary Table 2: Accumulation of cadmium in tissue of control and treated plants. Values are given in micrograms per gram of dry matter.

| Plant | Treatment | Cd accumulation in shoot [μg/g] | Cd accumulation in root [μg/g] |
| --- | --- | --- | --- |
| 1 | control | 0.92 | 1.79 |
| 2 | control | 0.86 | 1.57 |
| 3 | control | 0.75 | 1.88 |
| 4 | treated | 284.89 | 8883.63 |
| 5 | treated | 352.66 | 16904.59 |
| 6 | treated | 388.68 | 9115.51 |

#### Supplementary Table 3. List of qPCR primers.

| Cadmium in shoot | | | | |
| --- | --- | --- | --- | --- |
| Condition | Gene accession | Primers | Tm | Product size |
| Up regulated | HORVU.MOREX.r2.7HG0558200.1 | (F) CTAGAGACAACGAGGACCTGC | 59.9 | 80 |
|  |  | (R) CTCGGACGTGAGAGGGAAAC | 60.1 |  |
| Down regulated | HORVU.MOREX.r2.6HG0456470.1 | (F) GAGTGGGAGGAGCTCAACAG | 59.8 | 80 |
|  |  | (R) GCGAGGTTCAGAGAGATCCG | 60 |  |
| Control | HORVU.MOREX.r2.1HG0006620.1 | (F) TCAAGGCCTCCTCCATCTTC | 58.8 | 69 |
|  |  | (R) TCAGCGCGAGGTCTATCTTG | 59.6 |  |
| Cadmium in root | | | | |
| Up regulated | HORVU.MOREX.r2.2HG0162330.1 | (F) TGCTTGCTGTGGTGGTACTC | 60.3 | 80 |
|  |  | (R) GAAGTCCTGGAGCTGGCTTG | 60.7 |  |
| Down regulated | HORVU.MOREX.r2.7HG0538720.1 | (F) GTCAACGGAGCTGCGACTAC | 61.1 | 80 |
|  |  | (R) ATCTGCTTCGCTCCCAAGAC | 60.1 |  |
| Control | HORVU.MOREX.r2.7HG0561140.1 | (F) GCCTTGCAATGCCACAAATG | 59.2 | 70 |
|  |  | (R) TGCTGCAAATGAGCAAACCC | 60 |  |

#### Supplementary Table 4. List of primers for *HvPCR2* coding sequence amplification.

| Name | Sequence 5´->3´ |
| --- | --- |
| HvPCR2.1 F | ATGGCAACGCAGCAGTCGACGCG |
| HvPCR2.1 R | TCAACGGGTCATCCCTGGGTGC |
| HvPCR2.2 F | ATGAAGCCCGGCACCGAGCCGGCC |
| HvPCR2.2 R | TCAGCGGCCCATCTCCTGCATGCCT |
| HvPCR2.3 F | ATGAAGCCCGGCAGCGAGGCACCC |
| HvPCR2.3 F | CTAGCGGCCCATCTCCTGCATGCCG |
| HvPCR2.4 F | ATGAAGCCGGCGGCCCAGCCACTC |
| HvPCR2.4 R | CTAGCGGCCCATCTCCTGCATGCC |
| HvPCR2.5 F | TGAAGCCCGCCGCGCAGCCGG |
| HvPCR2.5 R | TTAGCGGCCCATCTCCTGTAC |

**Supplementary Table 5.** Percentage of amino acid identity between predicted HvPCR2 proteins and previously identified *Arabidopsis thaliana* PCR proteins.

|  | AtPCR1 | AtPCR2 | AtPCR3 | AtPCR11 | AtPCR4 | AtPCR5 | AtPCR6 | AtPCR7 | AtPCR9 | AtPCR8 | AtPCR10 |
| --- | --- | --- | --- | --- | --- | --- | --- | --- | --- | --- | --- |
| HvPCR2.1 | 33.47 | 36.29 | 35.08 | 33.07 | 29.64 | 27.27 | 33.07 | 28.46 | 27.60 | 25.10 | 21.56 |
| HvPCR2.2 | 44.24 | 49.40 | 48.80 | 47.27 | 37.31 | 33.85 | 32.00 | 40.63 | 40.74 | 29.53 | 28.22 |
| HvPCR2.3 | 46.45 | 53.85 | 51.28 | 47.47 | 37.89 | 33.51 | 30.80 | 43.33 | 40.65 | 28.65 | 27.86 |
| HvPCR2.4 | 45.06 | 53.37 | 48.47 | 49.38 | 36.13 | 32.80 | 30.67 | 41.56 | 38.99 | 28.50 | 27.72 |
| HvPCR2.5 | 45.96 | 52.47 | 48.15 | 49.69 | 35.79 | 32.98 | 31.25 | 41.67 | 39.87 | 27.80 | 28.86 |

Highest identity for each predicted HvPCR2 protein is highlighted in grey color.
